# Centralization reduces meniscal extrusion, improves joint mechanics and functional outcomes in patients undergoing meniscus surgery: A systematic review and meta‐analysis

**DOI:** 10.1002/ksa.12410

**Published:** 2024-08-09

**Authors:** Khalis Boksh, Duncan E.T. Shepherd, Daniel M. Espino, Arijit Ghosh, Randeep Aujla, Tarek Boutefnouchet

**Affiliations:** ^1^ Department of Biomedical Engineering University of Birmingham Birmingham UK; ^2^ Leicester Academic Knee Unit University Hospitals of Leicester NHS Trust Leicester UK; ^3^ Department of Trauma & Orthopaedics University Hospitals of Birmingham NHS Trust Birmingham UK

**Keywords:** centralization, meniscal extrusion, meniscal tears, meniscotibial ligament, osteoarthritis

## Abstract

**Purpose:**

To perform a systematic review and meta‐analysis of the existing literature on meniscal centralisation procedures, analysing its impact on meniscal extrusion, joint biomechanics and clinical and radiological outcome measures.

**Methods:**

The Cochrane Controlled Register of Trials, PubMed (MEDLINE) and Embase were used to perform a systematic review using the Preferred Reporting Items for Systematic Reviews and Meta‐Analyses criteria. Biomechanical studies on healthy animal or human cadaveric knee joints that assessed meniscal extrusion or tibiofemoral contact mechanics (contact area and pressure) following centralization for meniscal pathologies were included. For clinical studies, those that prospectively or retrospectively assessed patient‐reported outcome measures (PROMs), postoperative knee motion, complications and radiological extrusion following centralization for meniscal pathologies were included.

**Results:**

Fifteen studies were included in the analysis, comprising eight biomechanical, six clinical and one both. There were 92 knee specimens for biomechanical testing, of which 40 were human cadaveric and 52 porcine models. Biomechanical data revealed centralization to be commonly performed for posterior meniscal root tears and significantly reduced extrusion and contact pressure whilst improving contact area following a tear (*p* < 0.00001). Centralization restored extrusion to that of the native knee at all flexion angles described (0–90**°**, *p* = 0.25) and, compared to the torn state, brought tibiofemoral contact mechanics 3.2–5.0 times closer to the native state. Clinical data showed that 158 patients underwent centralization for extrusion. It improved postoperative Knee Injury and Osteoarthritis Outcome score (KOOS) (*p* = 0.006) and Lysholm scores (*p* < 0.00001) at 25.0 months, maintained extrusion reduction at 17.1 months (*p* < 0.00001) and preserved knee motion.

**Conclusion:**

Centralisation for various meniscal injuries associated with extrusion can reduce meniscal extrusion and improve joint biomechanics, along with clinical and radiological outcomes. Existing evidence is still scarce and exhibits a notable amount of methodological heterogeneity.

**Level of Evidence:**

Systematic review of Level IV evidence.

**Abbreviations**
ACL: anterior cruciate ligament
ATPR: anatomical transtibial pull through repair
BM: biomechanical
C‐Ad: centralization with advancement
FWB: full weight bearing
ICRS: International Cartilage Repair Society
IKDC: International Knee Documentation Committee
KA: knotless anchor
KL: Kellgren and Lawrence
KOOS: Knee Injury and Osteoarthritis Outcome Score
KSS: Knee Society Score
LCL: lateral collateral ligament
LFA: low flexion angle
LJSW: lateral joint space width
LM: lateral meniscus
LMD: lateral meniscus defect
LMPRT: lateral meniscus posterior root tear
LMTL: lateral meniscotibial ligament
LTP: lateral tibial plateau
MCL: medial collateral ligament
MCMS: Modified Coleman Methodology Score
MFL: meniscofemoral ligament
MHFA: mid‐to‐high flexion angles
MM: medial meniscus
MMPRT: medial meniscus posterior root tear
MMTL: medial meniscotibial ligament
MRI: magnetic resonance imaging
MT: meniscotibial
MTL: meniscotibial ligament
MTP: medial tibial plateau
MTS: medial tibial spine
NA: not applicable
NATPR: nonanatomical transtibial pull through repair
NR: not reported
OA: osteoarthritis
OB: Outerbridge
OR: odds ratio
OWHTO: open wedge high tibia osteotomy
PA: partial meniscectomy
PCL: posterior cruciate ligament
PH: posterior horn
PMRT: posterior meniscal root tear
PRISMA: Preferred Reporting Items for Systematic Reviews and Meta‐Analyses
PROMs: patient‐reported outcome measures
QOL: quality of life
ROM: range of motion
RTA: return to full activity
SAR: suture‐anchor repair
SMD: standard mean difference
TPR: transtibial pull‐through repair
VAS: visual analog scale

## INTRODUCTION

A recent consensus statement of leading experts suggests that meniscal extrusion [18], a strong risk factor for osteoarthritis [18, 21, 43], is often observed after posterior meniscal root tears (PMRT) [18, 22], radial tears [18, 62], meniscectomies [2, 18], saucerization of discoid menisci and transplantation [18, 25, 46, 59]. PMRTs are the most common, and despite numerous studies reporting success with the transtibial pull‐through repair technique (TPR) [9, 37, 54], postoperative extrusion can persist [36, 57]. Furthermore, such root tears are often diagnosed long after they occur [29, 52]. Where possible, meniscal repair should be preferred over resection to preserve meniscal function. However, not all tears are reparable and will end up requiring a partial or total meniscectomy [26]. Unfortunately, this can loosen the meniscocapsular attachment and cause extrusion [13, 24]. Based on these implications, a focus on surgical techniques to reduce meniscal extrusion has recently emerged.

One technique includes centralization. This is the process of re‐attaching the meniscal midbody‐capsular complex just central to the peripheral rim of the tibial plateau [40]. This can be through the insertion of either knotted or knotless suture anchors [6, 29, 31, 40], or by a transtibial stitch through a tunnel and tied over a button [14, 15]. In theory, by returning the meniscus to its natural position, its vital function of load distribution could be restored, helping to prevent cartilage degeneration after surgery [22, 49]. Ultimately, this would be beneficial in delaying the progression of osteoarthritis, and in providing surgeons an alternative treatment option for meniscal preservation prior to the introduction of more invasive approaches. However, it remains unclear whether such techniques can indeed improve joint biomechanics and, if so, whether this translates to improved clinical and radiological outcomes. Thus, a systematic review and meta‐analysis of the existing literature on centralisation techniques in meniscus surgery was performed. Our hypothesis was that it would improve these outcome measures, representing a promising approach to addressing knee joint dysfunction resulting from meniscal extrusion.

## METHODS

### Literature search

The Preferred Reporting Items for Systematic Reviews and Meta‐Analyses guidelines were used to find articles in PubMed (MEDLINE), Cochrane Central Register of Controlled Trials, and Embase [42]. Searches were conducted from the inception of the databases to 10 December 2023, and repeated on 14 July 2024 for an update of the literature. The search terms included the following: (‘centralization’ OR ‘peripheral stabilization’ OR ‘extrusion correction’ OR ‘meniscotibial repair’) AND (‘meniscus extrusion’ OR ‘meniscus’ OR ‘knee’) and are illustrated in Supporting Information S1: Online Resource S1. No restrictions were made on the date of publication or language, and efforts were made to obtain translated versions of all included studies. Restrictions were not placed on the date of publication or the journal. All relevant articles and reviews were examined for further relevant citations.

### Eligibility criteria and outcome measures

a. Biomechanical studies:
  Studies were included on healthy animal or human cadaveric knee joints that assessed meniscal extrusion or tibiofemoral contact mechanics (contact area and pressure) following centralization for meniscal pathologies.
  We excluded any biomechanical study in which these primary objectives were not investigated. Furthermore, any study where specimens had pre‐existing chondral or meniscal lesions, bony abnormalities, previous surgeries, or fractures were also excluded due to their role as potential confounders.
b. Clinical studies:
  Studies which clinically assessed patient‐reported outcome measures (PROMs), postoperative knee motion, complications and radiological extrusion following centralization for meniscal pathologies were also included.
  We excluded case reports, review articles, expert opinions, technical tips and publications pertaining to surgical techniques.

### Study selection and the assessment of quality of studies

Two authors (Khalis Boksh and Arijit Ghosh) independently reviewed the titles and abstracts from the search results, after which potentially suitable papers were reviewed in full by each author independently. This was undertaken using a Modified Coleman Methodological Score (MCMS), modified to account for the subject matter, with scores designated as follows: excellent (>85), good (70–84), fair (55–69) and poor (<55). These have been described in previous reports (Supporting Information S1: Online Resource S2) [4, 5, 10]. Any disagreement between the two authors, from screening to eligibility of the included studies, was highlighted to the two senior authors (Randeep Aujla and Tarek Boutefnouchet), and an agreement was reached based on the majority vote. The same two senior authors also performed the MCMS for studies in which there was more than a four‐point discrepancy between the two primary reviewers when assessing the methodological quality. A consensus was reached in which the average of all four reviewers' scores was selected.

The risk of bias in the included clinical studies was assessed and reported by the same two authors in accordance with the Risk of Bias in Nonrandomized Studies of Interventions tool [56]. Each item was judged according to high, moderate, low or unclear risk of bias.

### Data synthesis and statistical analysis

Review Manager (RevMan) software (version 5.4, Cochrane Training) was used for data synthesis. Odds ratios (ORs) were used for all dichotomous variables, and standard mean differences (SMD) for continuous parameters. Statistical heterogeneity was assessed using the *I* ^2^ and chi‐square tests. A *p*‐value < 0.1 and an *I* ^2^ > 50% were considered suggestive of statistical heterogeneity, prompting a random‐effects model. Otherwise, a fixed‐effects model was used.

## RESULTS

### Included studies

**Figure 1:**
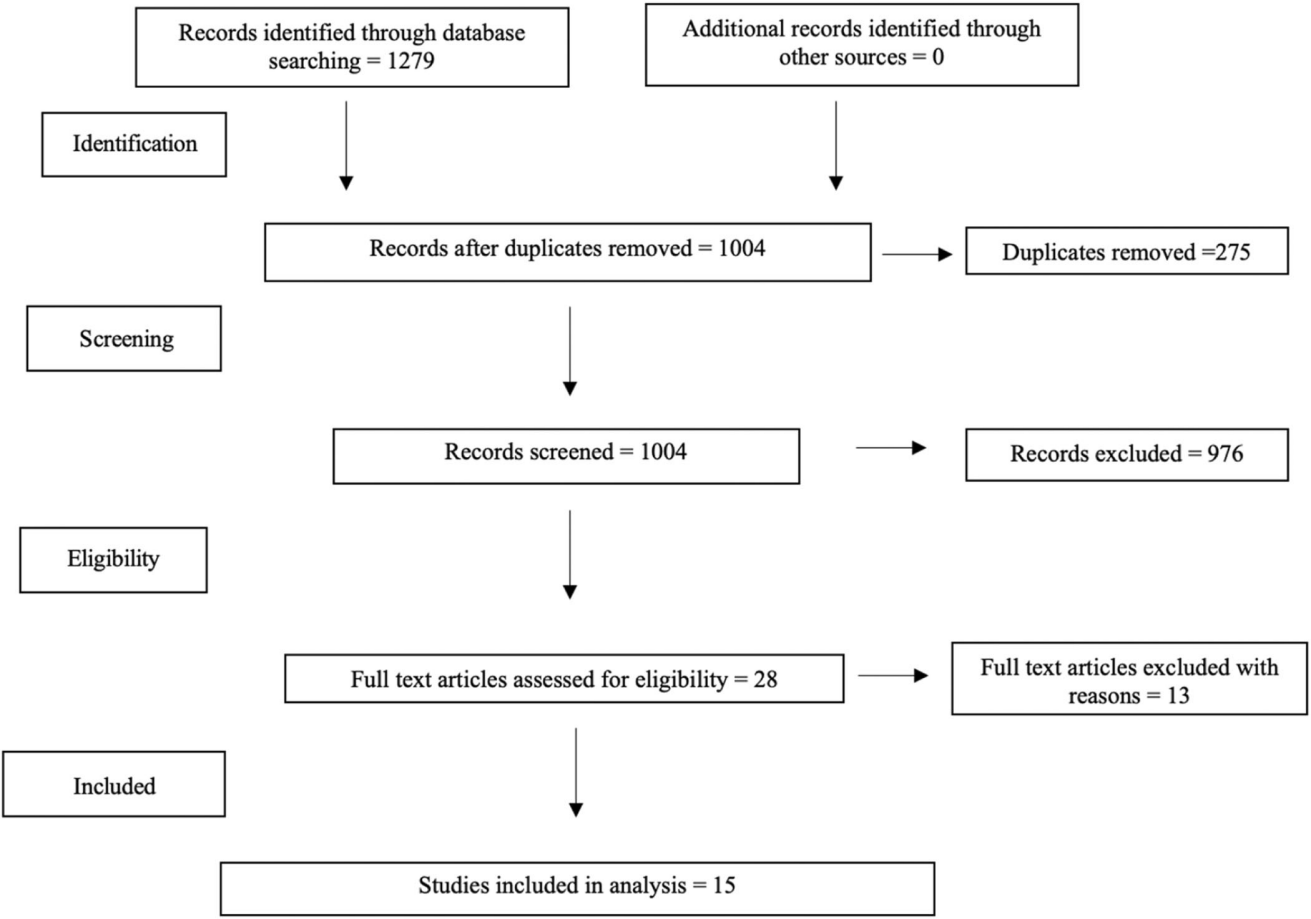
PRISMA flow diagram for study selection.

Twenty‐eight full texts were reviewed. Thirteen studies were excluded based on the eligibility criteria (Figure 1). Fifteen studies were therefore included in this systematic review [1, 14, 16, 23, 28, 30, 33, 35, 38, 44, 45, 48, 50, 58, 61].

### Biomechanical characteristics

Nine of the fifteen included studies provided biomechanical data. There were 92 testing specimens, of which 40 were in human cadaveric and 52 in porcine knees. The characteristics are described in Table 1.

**Table 1:** Biomechanical characteristics of the include centralization studies.

Study	Biomechanical comparison	Knee specimen (*n*)	Exclusion criteria	Experimental set up		Outcome measure		Surgical technique		
				Structure preservation	Load on biomechanical testing	Meniscal extrusion (mm)	Contact area and pressure	Root tear	Root repair	Centralization technique
Daney et al. [14]	Intact MM, MMPRT, ATPR ± centralization for MMPRT and NATPR ± centralization for MMPRT	Human Cadaveric (10)	Damaged menisci, ligament tears and >Grade 1 OB	All knee‐stabilizers	1000 N: 0°, 30°,60° and 90°	Distance between posterior MM and posterior border MCL Measured with 3D device	Tekscan	Transect adjacent to bony root attachment site	ATPR/NATPR	Transtibial stitch
Ozeki et al. [48]	Intact LM, LMPRT and centralization of meniscus with LMPRT	Porcine (6)	Damaged menisci and cartilage	MCL, ACL, PCL and MM. LCL cut for sensor insertion	200 N: 45°	Distance between 2 markers: PCL tibial attachment and LM outer edge	Tekscan	Transect 1 cm width LMPR	N/A	Suture anchor repair (Knotted)
Kubota et al. [38]	Intact LM, LMPRT and centralization of meniscus with LMPRT	Porcine (8)	Damaged menisci and cartilage	As per Ozeki [48]	200 N: 30°, 45°, 60° and 90°	As per Ozeki [48]	Tekscan	As per Ozeki [50]	N/A	Suture anchor repair (Knotted)
Paletta et al. [50]	Intact MMTL, torn MMTL and centralization of MMTL	Human Cadaveric (6)	Meniscal tear, advanced OA, cruciate injury	No dissection	1. Cyclical loading flexion‐extension at 0.2 Hz for 100 cycles 2. 10 nm varus load at 0°	Distance between MTP edge and MM outer edge Measured with US	N/A	N/A	N/A	Suture anchor repair (Knotless)
Debieux et al. [16]	Intact MM, 2, 3 and 4 mm extrusion, maximum extrusion and centralization	Human cadaveric (10)	Chondral or meniscal lesions	All knee‐stabilizers	1000 N: 0°, 30°, 60° and 90°	Distance between the edge of MTS edge and MTP Measured using a digital calliper	Tekscan	N/A	N/A	Suture anchor repair (knotted)
Kohno et al. [33]	Intact LM PM, PM with LMPRT, various centralization techniques for PM with LMPRT (1‐anchor, 2‐anchor and C‐Ad centralization)	Porcine (6)	Damaged menisci and cartilage	As per Ozeki [50]	200 N: 45°	N/A	Tekscan	Transect 1 cm width LMPR and MT ligament	N/A	Suture anchor repair (knotted)
Amano et al. [1]	Intact MM, MMPRT, NATPR alone, and various centralization techniques + NATPR for MMPRT (2 knotless and 3 knotless anchor centralization)	Porcine (10)	Damaged menisci and cartilage	ACL, PCL, LCL and MTL MCL cut for sensor insertion	200 N: 30°, 45°, 60° and 90°	Distance between 2 markers: posterior border of MCL and posterior MM	Tekscan	Transect 9 mm medial to MMPR attachment	NATPR	Suture anchor repair (knotless)
Ueki et al. [58]	ACLR + intact MM, ACLR + MMPRT, ACLR + centralization of MMPRT	Human Cadaveric (14)	Meniscal damage, ligament tears and KL > 2	All knee‐stabilizers	89 N: 0° and 30°	As per Paletta Jr [50]	Tekscan	Transect 1 cm width MMPR	N/A	Suture anchor repair (knotted)
Morales‐Avalos et al. [45]	Intact LM, LMTL injury, MFL injury, capsulodesis for LMTL and centralization for LMTL and MFL	Porcine (22)	Previous surgeries or fractures. Lateral meniscal injury	All knee‐stabilizers	200 N: 30° and 60°	As per Ozeki [48]	N/A	N/A	N/A	Suture anchor repair (knotted)

Abbreviations: ACL, anterior cruciate ligament; ACLR, anterior cruciate ligament reconstruction ARTPR, anatomical transtibial pull through repair; C‐Ad, centralization with advancement; KL, Kellgren‐Lawrence grading; LCL, lateral collateral ligament; LM, lateral meniscus; LMPR, lateral meniscus posterior root; LMPRT, lateral meniscus posterior root tear; LMTL, lateral meniscotibial ligament; MCL, medial collateral ligament; MFL, meniscofemoral ligament; MM, medial meniscus; MMPR, medial meniscus posterior root; MMPRT, medial meniscus posterior root tear; MMTL, medial meniscotibial ligament; MT, meniscotibial; MTP, medial tibial plateau; MTS, medial tibial spine; N/A, not applicable; NATPR, nonanatomical transtibial pull through repair; OA, osteoarthritis; OB, outerbridge; PCL, posterior cruciate ligament; PM, partial meniscectomy; US, ultrasound.

Specimens were prepared similarly across all studies. The majority tested the specimens at a range of flexion angles (0–90°) [1, 14, 16, 38, 45, 58]. To conduct a meta‐analysis, certain factors were taken into consideration. First, all types of centralization techniques in each study were explored. Second, given the influence range of motion has on load distribution in the meniscus [60], subgroups of low (LFA) (0°–45°) and mid‐to‐high flexion angles (MHFA) (60°–90°) were created. Based on these points, certain studies were used more than once, given their wealth of data. Third, it was ensured that outcomes were measured with precise means and similar metrics and that various causes of extrusion were unpooled to minimize heterogeneity. The key tibiofemoral contact mechanics measured included average contact pressure at the tibial cartilage and contact area at the meniscal body.

### Clinical characteristics

The mean MCMS score of the seven included clinical studies was 61.7. Their characteristics are described in Table 2.

**Table 2:** Baseline characteristics of clinical studies.

Study	Study design	Mean age (range/SD)	Gender (male,%)	Cause of extrusion (%)	Study participants (%)		Centralization		Postoperative rehabilitation	Mean follow‐up (months)	Outcomes	MCMS	Overall quality of study
					No centralization (Isolated meniscal root repair, capsulodesis, meniscectomy)	Centralization	Technique	Description					
Koga et al. [28]	Prospective series	29 (13–53)	11 (52.4)	Isolated LM defect (40) Saucerization discoid LM (60)	0 (0)	20 (100)	Knotted SAR	2 JuggerKnot anchors edge of LTP. Sutures from both anchors passed through capsule and LM centralized via sliding knots	Day 0: Full ROM FWB: 0–4/52 RTA: 4/12	24	ROM, Lysholm, KOOS, LJSW, LME on coronal MRI (12 months)	69	Fair
Paletta Jr et al. [50]	Retrospective series	55 ± 9	4 (26.7)	Medial meniscotibial ligament disruption (100)	0 (0)	15 (100)	Knotless SAR	See Table 1	NR	3.5 ± 1.88	Pre‐ and post‐op MME via USS	41	Poor
Koga et al. [30]	Prospective series	43 (14–58)	12 (44.4)	Lateral meniscal defect (100): Subtotal lateral meniscectomy (74.1), OA (25.9)	0 (0)	27 (100)	Knotted SAR	As per Koga [28] but with additional capsular advancement to reform meniscus shape	Day 0: Full ROM FWB: 0–4/52 RTA: 6/12	24	As per Koga 2016 [28] IKDC	69	Fair
Mochizuki et al. [44]	Prospective series	62.1 ± 6.0	8 (30.8)	MMPRT (100)	0 (0)	26 (100)	Knotted SAR	Technique as per Koga [28] for MM	Day 0: 20° knee lock FWB: 4/52 RTA: 6/12	35.4 ± 12.9	Lysholm, KOOS. MME on coronal MRI	64	Fair
Katagiri et al. [23]	Retrospective Cohort	60.2 (45.2–72.2)	12 (34.3)	Medial OA (100)	OWHTO: 14 (40)	OWHTO + Centralization: 21 (60)	Knotted SAR	Technique as per Koga [28] for MM	Day 0: Full ROM FWB at 14 days RTA: 6/12	31.9 ± 8.1	Lysholm, IKDC, KOOS. MJSW	76	Good
Krych et al. [35]	Prospective series	50.1 ± 11.3	6 (24)	MMPRT (100)	0 (0)	25 (100)	Knotless SAR	2–3 Knotless FiberTak (Arthrex) at posteromedial MM body Sutures of each anchor passed through capsule and MM centralized with tensioning	Day 0: Full ROM FWB: 4–6/52 RTA: 8–16/52	24 ± 7.2	KOOS Jr, IKDC. MME on coronal MRI (6 months)	66	Fair
Wang et al. [61]	Retrospective series	56 (46–53)	11 (45.8)	Medial OA (100)	0 (0)	OWHTO + Centralization: 24 (100)	Knotless SAR	2 anchors edge of MTP technique as per Krych [35]	NR	24	Knee society score	47	Poor

Abbreviations: FWB, full weightbearing; ICRS, International Cartilage Repair Society; IKDC, International Knee Documentation Score; KOOS, Knee Injury and Osteoarthritis Outcome Score; LJSW, lateral joint space width; LM, lateral meniscus; LME, lateral meniscal extrusion; LTP, lateral tibial plateau; MJSW, medial joint space width; MM, medial meniscus; MME, medial meniscus extrusion; MMPRT, medial meniscus posterior root tear; MRI, Magnetic Resonance Imaging; MTP, medial tibial plateau; NR, not reported; OA, Osteoarthritis; OWHTO, Open Wedge High Tibial Osteotomy; ROM, range of motion; RTA, return to full activity; SAR, suture anchor repair; USS, Ultrasound Scan; VAS, Visual Analogue Scale.

One hundred and fifty‐eight patients (mean age, 50.9 years) underwent centralization for meniscal extrusion. Meniscal extrusion was caused by medial meniscus posterior root tears (MMPRT) in 51 patients (32.3%) [35, 44], lateral meniscal defect (LMD) secondary to meniscectomy or saucerization for discoid menisci in 40 patients (25.3%) [28, 30], isolated meniscotibial ligament disruption in 15 patients (9.5%) [50] and mild‐to‐moderate OA in 52 patients (*n* = 32.9%) [23, 61].

To conduct a meta‐analysis between pre‐ and postoperative changes, we ensured certain outcomes were measured in ≥3 studies. The most common patient‐reported outcomes measured were the Lysholm and Knee Injury and Osteoarthritis Outcome Score (KOOS) [28, 30, 44]. Regarding meniscal extrusion, four studies provided similar metrics to conduct a meta‐analysis [28, 30, 44, 50].

### Study risk of bias assessment

**Figure 2:**
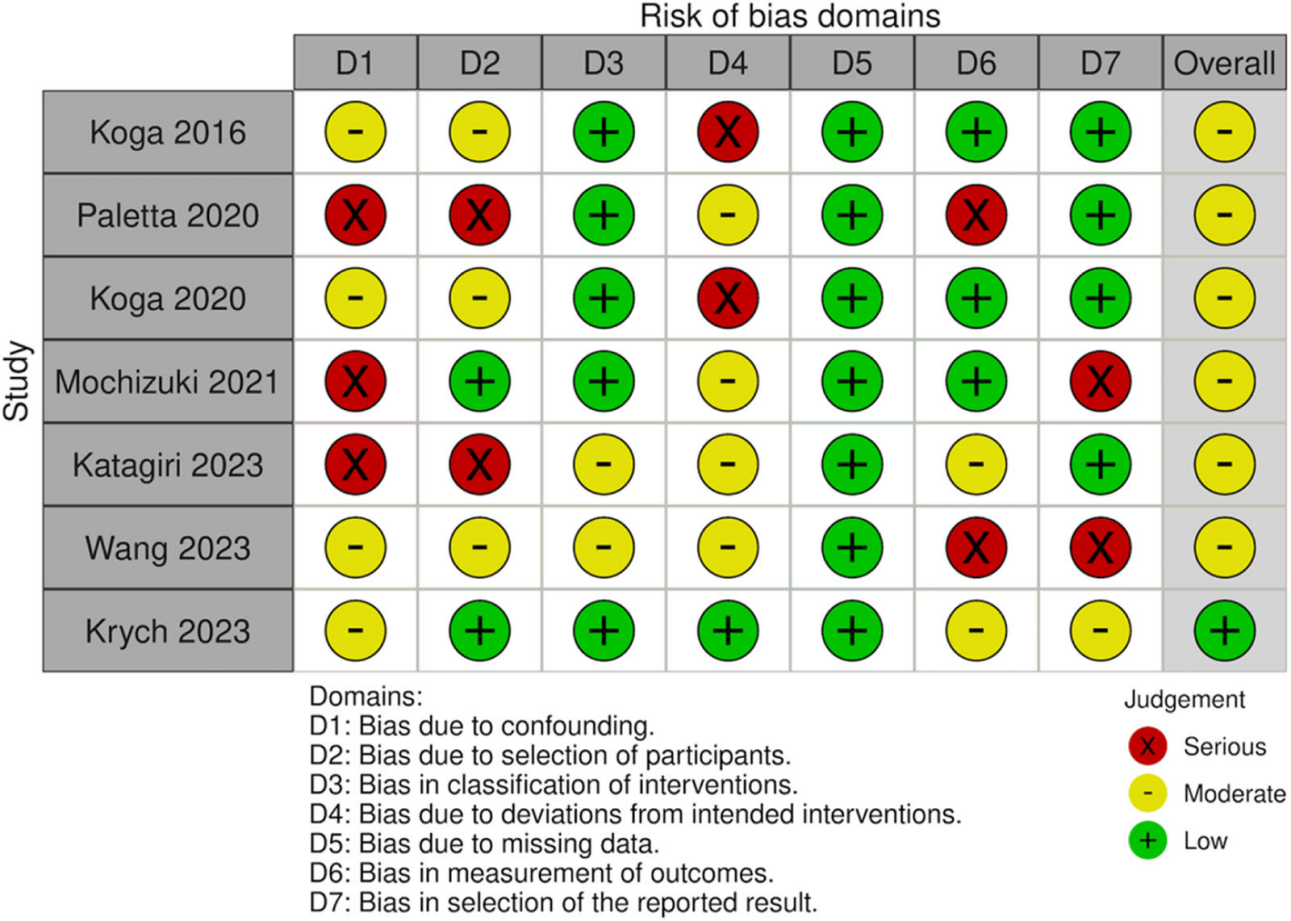
Risk of bias summary. The ‘traffic light’ plots the domain‐level judgements for each individual result. There are seven domains (D) which include the following: D1: Bias due to confounding data (selection bias), D2: bias in selection of participants into the study (selection bias), D3: bias in classification of interventions (information bias), D4: bias due to deviations from intended interventions (performance bias), D5: bias due to missing data (attrition data), D6: bias in measurement of outcomes (detection bias), and D7: bias in selection of the reported result (outcome reporting bias). Each domain for each study is classified as either high risk of bias (red circle), moderate risk of bias (yellow circle) or low risk of bias (green circle).

**Figure 3:**
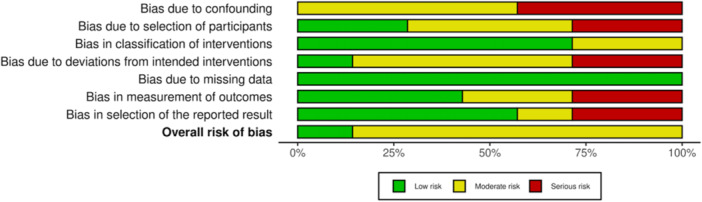
Risk of Bias graph. Within each bias domain, weighted bars are plotted of the distribution of the risk of bias judgements across all included studies.

Given the nonrandomized design of all studies, the risk of selection and performance bias was moderate to high. The risk of all other biases was generally low to moderate (Figures 2 and 3).

## BIOMECHANICAL ANALYSES

Data for meniscal root tears are presented as forest plots. Qualitative data can be found in Supporting Information S1: Online Resources S3–S5, respectively.

a.
**Meniscal root tears**

### Meniscal extrusion

**Figure 4:**
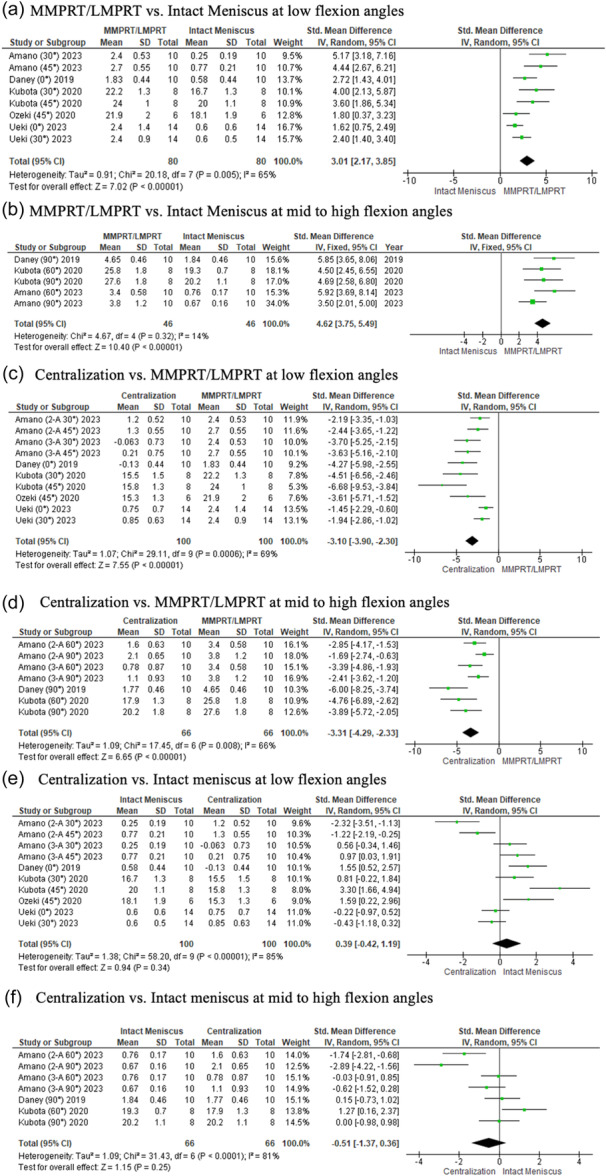
Forest plots of the comparison between intact meniscus, MMPRT/LMPRT and centralization for meniscal extrusion (mm) at low and mid to high flexion angles. 2‐A, 2 anchor; 3‐A, 3 anchor; C‐Ad, centralization with advancement; CI, confidence intervals IV, inverse variance; LMPRT, lateral meniscus posterior root tear; MMPRT, medial meniscus posterior root tear; SD, standard deviation.

At all flexion angles, meniscal extrusion was significantly greater in the presence of a medial or lateral meniscus posterior root tear compared to the intact state (LFA, *p* < 0.00001; MHFA *p* < 0.0001) (Figure 4a,b). Centralization significantly reduced meniscal extrusion from the torn state (LFA, *p* < 0.00001; MHFA, *p* < 0.00001), to values similar to the intact state at all angles (LFA, *p* = 0.34; MHFA, *p* = 0.25) (Figure 4c–f).

### Average contact pressure

**Figure 5:**
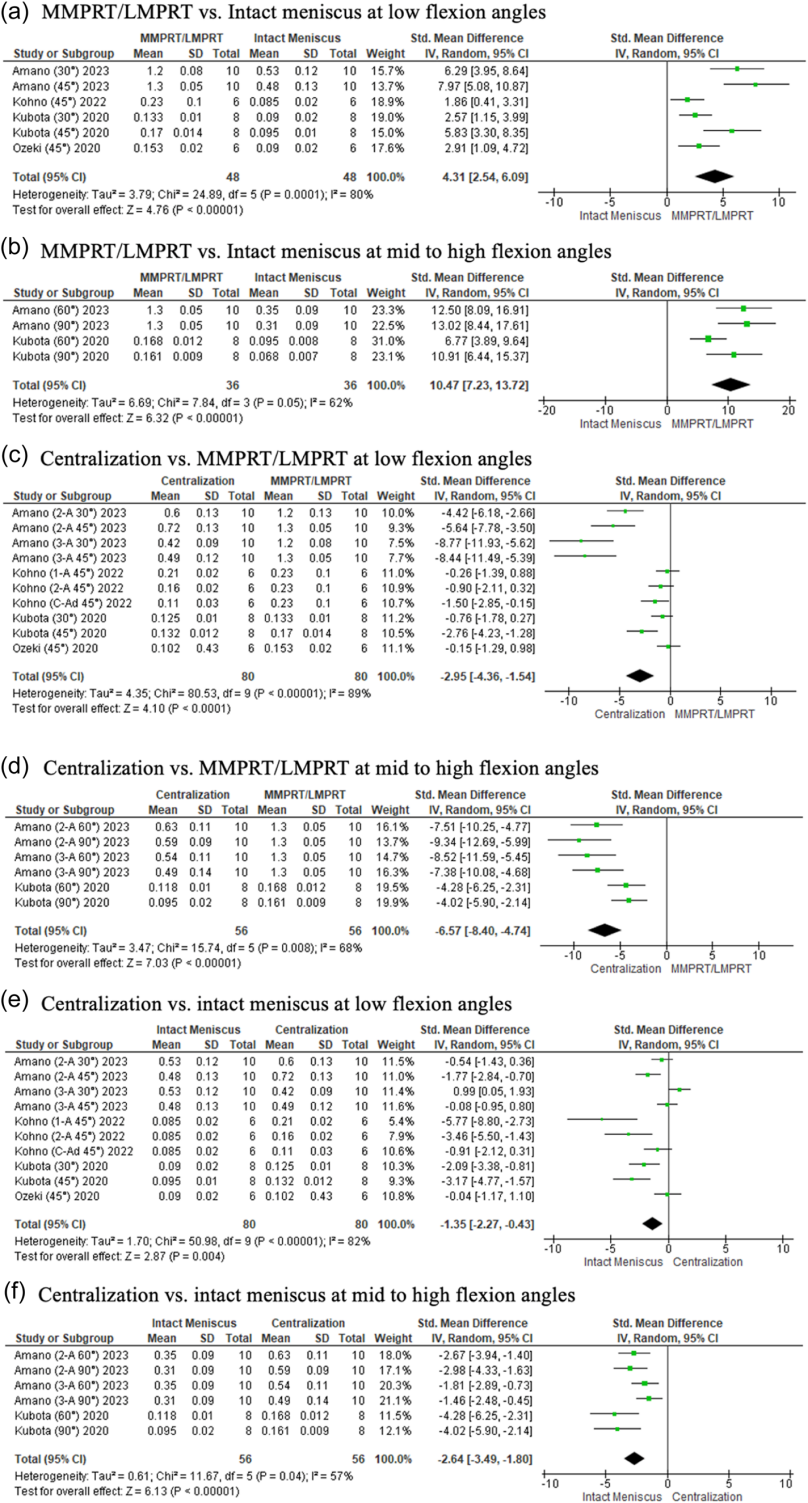
Forest plots of the comparison between intact meniscus, MMPRT/LMPRT and centralization for average contact pressure at the tibial cartilage (MPa) at low and mid to high flexion angles. 2‐A, 2 anchor; 3‐A, 3 anchor; C‐Ad, centralization with advancement; CI, confidence intervals IV, inverse variance; LMPRT, lateral meniscus posterior root tear; MMPRT, medial meniscus posterior root tear; SD, standard deviation.

At all flexion angles, significantly higher contact pressure at the tibial cartilage occurred in the torn state compared to the intact state (LFA, *p* < 0.00001; MHFA, *p* < 0.00001) (Figure 5a,b)*.* Centralization of the torn condition significantly reduced the contact pressure (LFA, *p* < 0.0001; MHFA, *p* < 0.00001) (Figure 5c,d). However, it did not restore the normal distribution of load, with significantly lower pressure observed in the intact state (LFA, *p* = 0.004; MHFA, *p* < 0.00001) (Figure 5e–f).

### Average contact area

**Figure 6:**
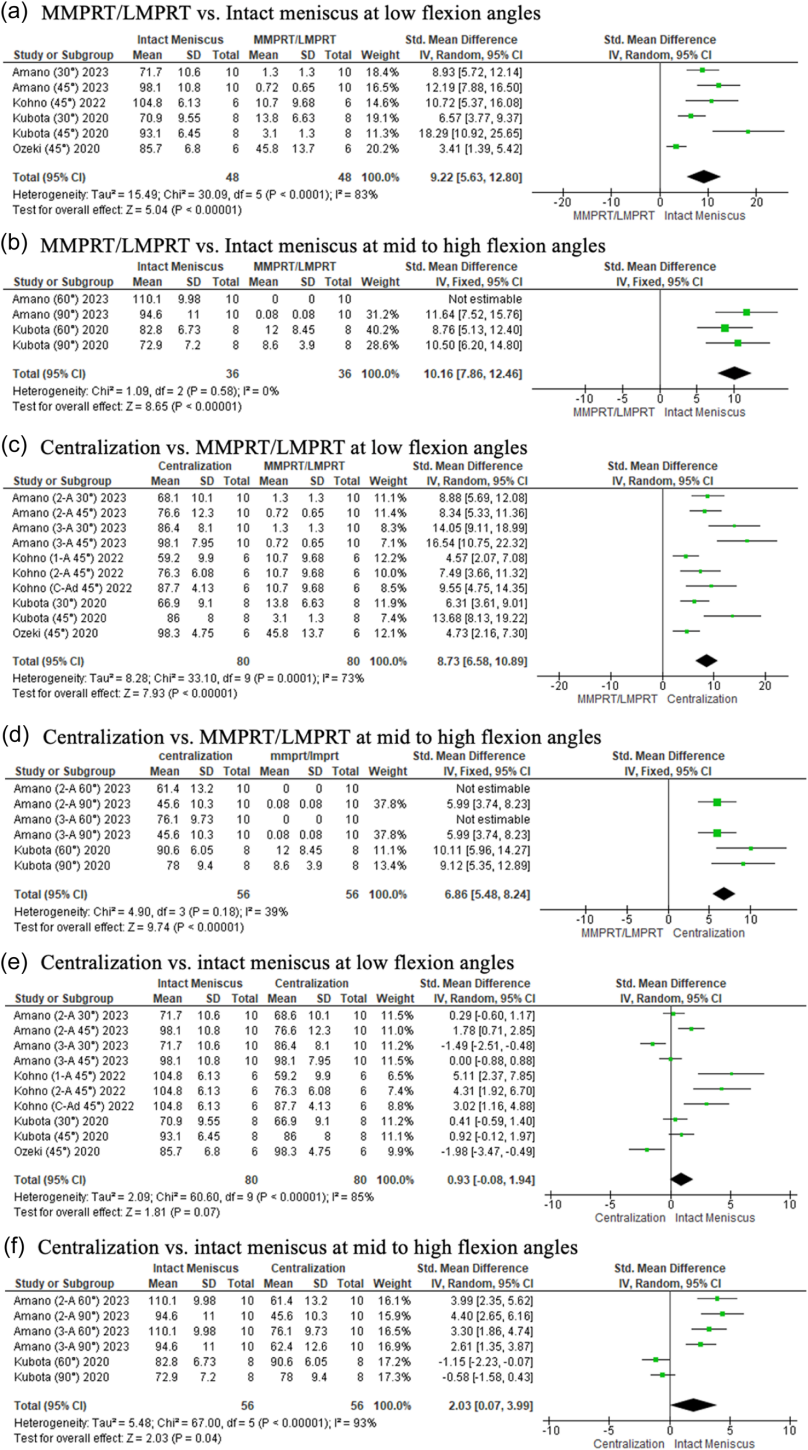
Forest plots of the comparison between intact meniscus, MMPRT/LMPRT and centralization for average contact area at the body of the meniscus (mm^2^) at low and mid to high flexion angles. 2‐A, 2 anchor; 3‐A, 3 anchor; C‐Ad, centralization with advancement; CI, confidence intervals IV, inverse variance; LMPRT, lateral meniscus posterior root tear; MMPRT, medial meniscus posterior root tear; SD, standard deviation.

At all flexion angles, the average contact area of the meniscus was greater in the intact than the torn state (LFA, *p* < 0.00001. MHFA, *p* < 0.00001) (Figure 6a,b)*.* Centralization of the torn meniscal root significantly improved the average contact area (LFA, *p* < 0.00001; MHFA, *p* < 0.00001) (Figure 6c,d). However, it only restored the distribution of the load to that of the intact state at lower flexion angles (LFA, *p* = 0.07; MHFA, *p* = 0.04) (Figure 6e,f).

b.
**Meniscotibial ligament injury** (Table 3 **)**

**Table 3:** Qualitative synthesis and subgroup analysis of centralization techniques in patients with meniscotibial ligament injury or meniscal root repair.

Study	Pathology/treatment	Meniscal extrusion (mm)				Average contact pressure (MPa)			Average contact area (mm^2^)		
		Centralization versus MTL injury	Centralization versus intact	Root repair versus intact	Root repair versus root repair with centralization	Centralization versus MTL injury	Centralization versus intact	Root repair versus root repair with centralization	Centralization versus MTL injury	Centralization versus intact	Root repair versus root repair with centralization
Daney et al. [14]	MMPRT Repair	N/A	**0°**: –0.13 ± 0.44–0.58 ± 0.44, *p* > 0.05 **90°**: 1.77 ± 0.46–1.84 ± 0.46, *p* > 0.05	**0°**: 0.70 ± 0.44–0.58 ± 0.44, *p* > 0.05 **90°**: 2.41 ± 0.46–1.84 ± 0.46, *p* > 0.05	**0°**: 0.70 ± 0.44 to −0.13 ± 0.44, *p* > 0.05 **90°**: 2.41 ± 0.46–1.77 ± 0.46, *p* > 0.05	N/A	*p* > 0.05 all angles	*p* > 0.05 all angles	N/A	*p* > 0.05 at 0, 30 and 90°	*p* > 0.05 all angles
Paletta Jr et al. [50]	MMTL	**0°:** 2.1 ± 0.4 < 3.4 ± 0.7, *p* < 0.05	**0°**: 2.1 ± 0.4 versus 1.5 ± 0.6, *p* > 0.05	N/A	N/A	NR	NR	N/A	NR	NR	N/A
Debieux et al. [16]	MMTL	NR	*p* > 0.05	N/A	N/A	*p* > 0.05	*p* > 0.05	N/A	**0°**: 427 ± 144–360 ± 146, *p* > 0.05 **30°**: 425 ± 157 > 385 ± 140, *p* < 0.05 **60°**: 380 ± 140 > 304 ± 127, *p* < 0.05 **90°**: 283 ± 148–238 ± 148, *p* > 0.05	**0°**: 427 ± 144–456 ± 102, *p* > 0.05 **30°**: 425 ± 157–544 ± 120, *p* > 0.05 **60°**: 380 ± 140–429 ± 125, *p* > 0.05 **90°**: 283 ± 148 < 382 ± 124, *p* < 0.05	N/A
Amano et al. [1]	MMPRT Repair	NA	**3‐anchor** **30°**: 0.06 ± 0.73–0.25 ± 0.19, *p* > 0.05 **45°**: 0.21 ± 0.75–0.77 ± 0.21, *p* > 0.05 **60°**: 0.78 ± 0.87–0.76 ± 0.17, *p* > 0.05 **90°**: 1.1 ± 0.93–0.67 ± 0.16, *p* > 0.05	**30°**:1.5 ± 0.47–0.25 ± 0.19, *p* > 0.05 **45°**: 1.7 ± 0.46–0.77 ± 0.21, *p* > 0.05 **60°**: 2.3 ± 0.4–0.76 ± 0.17, *p* > 0.05 **90°**: 2.2 ± 0.53–0.67 ± 0.16, *p* > 0.05	**NATPR versus NATPR** + **3‐anchor** **30°:** 1.5 ± 0.47 > −0.063 ± 0.73 *p* < 0.05 **45°:** 1.7 ± 0.46 > 0.21 ± 0.75, *p* < 0.05 **60°:** 2.3 ± 0.4 > 0.78 ± 0.87, *p* < 0.05 **90°:** 2.2 ± 0.53–1.1 ± 0.93, *p* > 0.05	N/A	NR	**NATPR versus NATPR** + **3‐anchor** **30°**: 0.83 ± 0.08 > 0.42 ± 0.09, *p* < 0.05 **45°**: 0.84 ± 0.12 > 0.49 ± 0.12, *p* < 0.05 **60°**: 0.74 ± 0.11 > 0.54 ± 0.11, *p* < 0.05 **90°**: 0.75 ± 0.10 > 0.49 ± 0.14, *p* < 0.05	N/A	**3‐anchor** **30°**: 86.4 ± 8.1–71.7 ± 10.6, *p* > 0.05 **45°**: 98.1 ± 7.95–98.1 ± 10.8, *p* > 0.05 **60°**: 76.1 ± 9.73–110.1 ± 9.98, *p* > 0.05 **90°**: 62.4 ± 12.6 < 94.6 ± 11, *p* < 0.05	**NATPR versus NATPR** + **3‐anchor** **30°**: 33.3 ± 6 < 86.4 ± 8.1, *p* < 0.05 **45°**: 35.1 ± 7 < 98.1 ± 7.95, *p* < 0.05 **60°**: 38.5 ± 6.73 < 76.1 ± 9.73, *p* < 0.05 **90°**:27.1 ± 7.95 < 62.4 ± 12.6, *p* < 0.05
Morales‐Avalos et al. [45]	LMTL	**30°**: 1.42 ± 0.1 < 2.11 ± 0.16, *p* < 0.05 **60°**: 1.47 ± 0.05 < 2.42 ± 0.17, *p* < 0.05	**30**°: 1.42 ± 0.1–1.66 ± 0.65, *p* > 0.05 **60°**: 1.47 ± 0.05–1.69 ± 0.07, *p* > 0.05	N/A	N/A	NR	NR	N/A	NR	NR	N/A

Abbreviations: LMTL, lateral meniscotibial ligament injury; MMPRT, medial meniscus posterior root tear; MMTL, medial meniscotibial ligament; MTL, meniscotibial ligament; N/A, not applicable; NATPR, nonanatomical transtibial pull‐through repair; NR, not reported.

### Meniscal extrusion

Qualitative data revealed centralization to significantly reduce meniscal extrusion from a meniscotibial ligament (MTL) injury/tear at low to mid‐flexion angles [45, 50], to values similar to the intact state [16, 45, 50].

### Average contact pressure and contact area

Of the three included studies, only one investigated contact pressure and contact area [16]. Regarding contact pressure, it was concluded that this is similar between centralization intact and injured states. However, no quantitative data was provided, nor was measurement taken at the tibial cartilage. Centralization significantly improved contact area following an MTL injury at mid‐flexion angles and this was fully restored to the intact state at all angles. However, no description was provided where the contact area was measured.

c.
**Pairwise comparisons in the presence of root repair** (Table 3 **)**

Two studies compared centralization in combination with a root repair versus root repair alone [1, 14]. Although both reported that a root repair alone would restore meniscal extrusion to the intact state at all angles, Amano et al reported the addition of a 3‐knotless anchor (KA) centralization to reduce extrusion further in most angles [1]. The same study also reported both a significant reduction in contact pressure and improvement in contact area at all flexion angles with the addition of the 3 KA technique compared to root repair alone. However, the second study showed no differences in these outcomes between the two testing states [14].

## CLINICAL AND RADIOLOGICAL OUTCOMES

Data on Lysholm and Knee Injury and Osteoarthritis Outcome scores (KOOS) (mean time to assessment, 25.0 months) and meniscal extrusion (mean time to assessment, 17.1 months) are presented as forest plots. Qualitative synthesis can be found in Supporting Information S1: Online Resource S6.

### Lysholm and KOOS scores

**Figure 7:**
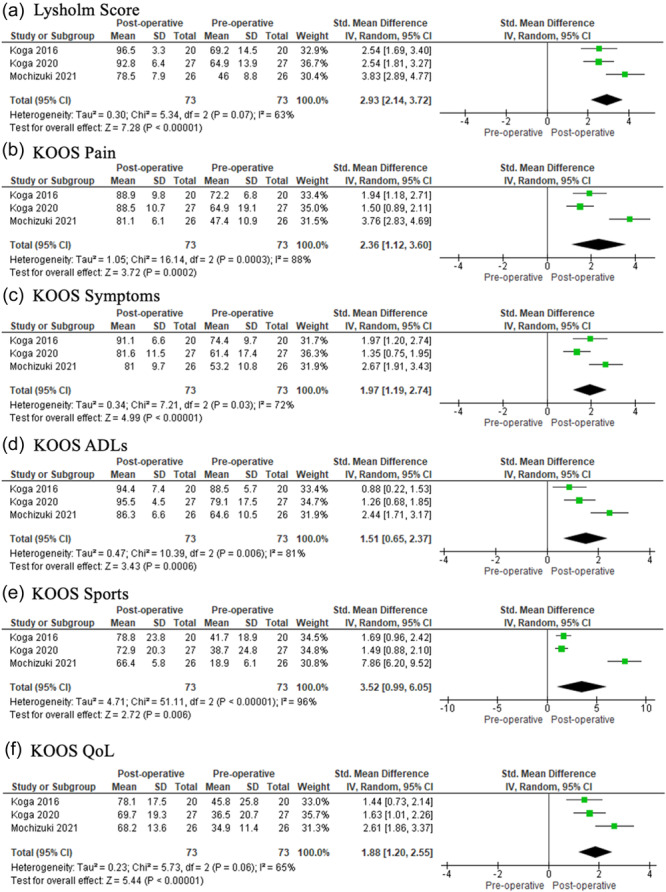
Forest plots of the comparison between pre‐ and postoperative Lysholm and Knee Injury and Osteoarthritis Outcome (KOOS) scores following centralization of the meniscus. CI, confidence interval; SD, standard deviation; IV, inverse variance.

Centralization led to significant improvement in all postoperative scores (Lysholm, *p* < 0.00001; KOOS pain, *p* = 0.0002; KOOS symptoms, *p* < 0.00001; KOOS Daily Living, *p* = 0.0006; KOOS Sports, *p* = 0.006; KOOS quality of life (QOL), *p* < 0.00001) (Figure 7a–f).

### Meniscal extrusion

**Figure 8:**
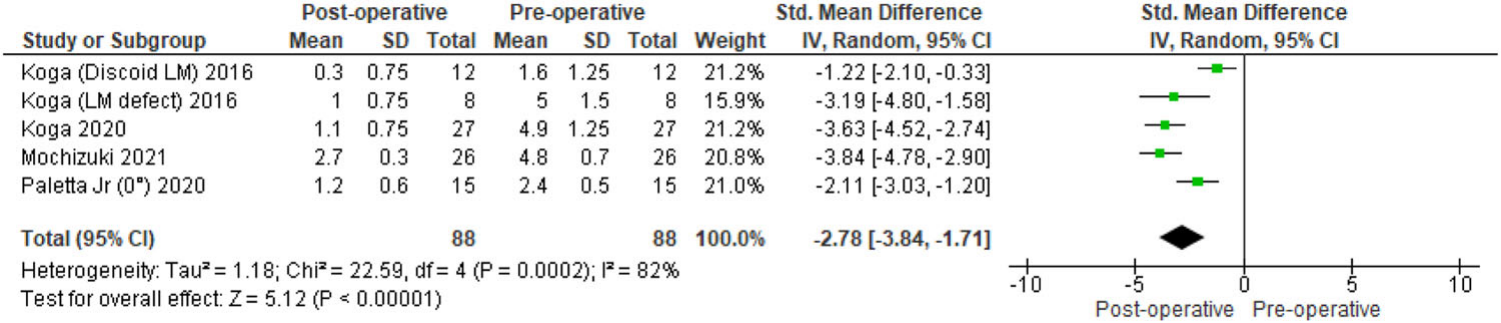
Forest plot of the comparison between pre‐ and postoperative changes in meniscal extrusion following centralization of the meniscus. CI, confidence interval; IV, inverse variance; LM, lateral meniscus; SD, standard deviation.

Postoperatively, centralization significantly reduced meniscal extrusion at a mean follow‐up of 17.1 months (SMD, −2.78; 95% confidence interval [CI], −3.84 to −1.71, *p* < 0.00001) (Figure 8).

A qualitative synthesis of other outcomes is presented in Supporting Information S1: Online Resource S7. Centralization significantly improved postoperative IKDC score [30, 35], and did not inhibit range of motion of the injured leg when compared to the contralateral side [28, 30]. The technique also improved postoperative joint width, especially compared to isolated OWHTO in the medial OA group [23].

## DISCUSSION

The most important findings of the present study were (i) meniscal centralisation effectively reduced meniscal extrusion and yielded improved tibiofemoral contact mechanics and functional outcomes for meniscal tears with a high risk of meniscal extrusion at short‐ to mid‐term follow‐up; (ii) centralisation was able to preserve knee ROM with few complications; (iii) in early OA, centralisation may play a role in joint space preservation whilst improving varus deformity in medial compartment disease.

Most of the biomechanical studies analysed the effects of centralization on meniscal root tears [1, 14, 33, 38, 48, 58]. In summary, at all angles, the meniscus was no longer displaced after centralization from its torn state, with extrusion values similar to the intact condition, indicating a successful reduction of the meniscus to its native position. The present study also shows that centralization techniques were able to significantly reduce the tibial contact pressure from its torn state. Although it was not able to fully restore tibial contact pressure distribution to that of the native state, it was able to bring it 3.2–4.0 times closer (Figure 5a,e; Low flexion angles: SMD, 4.36 × 1.35. Figure 5b,f; mid to high flexion angles: SMD, 10.47 × 2.64)] at all angles compared to the torn condition. Similar observations were made regarding its effect on contact area at mid‐to‐high flexion angles, bringing it five times closer (Figure 6b,f; mid to high flexion angles: SMD, 10.16 × 2.03) to the native state. These findings indicate the advantages centralization provides in a root‐torn knee, protecting the cartilage from excessive load and delaying cartilage degeneration [39, 49]. However, as all biomechanical studies were performed at time zero, the process of biological healing or potential loosening was unaccounted for. Furthermore, as root repair is the recommended treatment in restoring hoop stress function [3], with subgroup analysis revealing mixed results with and without centralization [1, 14], the technique should be considered as an adjunct only if concerns regarding persistent extrusion following root repair are present. Clinically, extrusion can persist despite a well‐performed root repair [36, 57]. Often, root tears are diagnosed longer after its onset and repair may be difficult due to degenerative changes [7, 22]. The purpose of centralization is to return the meniscus to its native position either by a suture anchor or transtibial stitch technique [6, 14, 15, 29, 31, 40]. Although this does not restore the meniscal hoop structure, a centralized meniscus can be chondroprotective [32, 49], and therefore, one should consider its use with root repair in patients with chronic tears.

Recent evidence suggests meniscal extrusion can occur with an isolated MTL [17, 22, 34]. Following the loss of the menisco‐capsular attachments, the meniscus is destabilized and dislodged from the tibial plateau. Costa et al. found extrusion greater than 3 mm to be linked to complex degenerative changes [12], with Lerer et al describing these changes to be as high as 69% [41]. Furthermore, Krych et al. found that they were more likely to be associated with an MTL injury [34]. The current review advocates the use of centralization in MTL injuries, in its ability to restore extrusion values to that of the uninjured knee [16, 27, 50].

Meniscal transplantation can restore meniscal function from total or subtotal meniscal volume loss either in isolation or combined with other joint preservation procedures [18, 25, 47, 60]. Despite the good outcomes [51], they are not widely available. Moreover, persistent extrusion after meniscal transplantation has been reported, potentially encouraging the development of OA [59]. However, the application of centralization techniques of the included studies showed significant improvement postoperatively in all PROMs measured, suggesting that meniscal centralisation is, at least, able to provide short‐term alleviation of symptoms.

One may suggest a peripheral centralization stitch to over‐constrain the knee, limiting normal excursion of either the medial or lateral menisci during knee movement. Of the included studies, no deficit was observed at the extreme ends of motion [28, 30]. However, the long‐term effects are unknown due to the relatively short follow‐up.

All but one clinical study measured extrusion with MRI. Although many studies have advocated its role in diagnosis [11, 19, 47], one main limitation of MRI includes the inability to fully determine the extent of dynamic extrusion in patients lying in supine, nonweightbearing positions [53]. More recently, there has been a trend in the use of ultrasound for diagnosing extrusion, particularly in the early stages and to guide diagnostic treatment of meniscal injuries [20]. Only two of the 15 included studies used ultrasound, and this is an area for future exploration.

## LIMITATIONS

The limitations of the present review stemmed largely from the quality of available evidence. Selection bias due to the retrospective or case series design in most of the studies may have influenced the results. The lack of control patients in these case series limited the ability to evaluate the extent to which postoperative outcomes are attributed to centralization itself, particularly in the studies where root repairs were performed. Furthermore, there was a relatively small number of patients in each study, and postoperative rehabilitation differed between groups which could potentially confound the data. A follow‐up period of 25 months is not long enough to identify cartilage degeneration or development of osteoarthritis, both of which can negatively affect patient‐reported outcome measures. Although the surgical procedures were similar between studies, the centralization techniques were not standardized, with transtibial pull‐out, knotted and knotless anchor repairs investigated. Although this can cause heterogeneity, the main objective of this review was to identify whether centralization has a role in meniscus surgery. Knotless anchors are a modified version of the knotted suture‐bridge technique [31]. In the context of the medial meniscus, the conventional technique can sometimes fail to reduce the most extruded location, at the posterior border of the MCL, owing to the difficulty in providing an anchor for the corresponding position from the mid‐medial portal [29, 31]. Using knotless anchors, where a posteromedial portal is positioned just below the MM, allows anchor fixation at this location whilst also increasing the contact area between the meniscocapsular attachments and the edge of the tibial plateau [31]. Theoretically, this can lead to a better reduction of extrusion.

Another limitation includes the differences in the specimens used between biomechanical studies, including human cadaveric and porcine knees. However, the latter is accepted as a reasonable surrogate in compressive load experiments [8, 55]. Furthermore, an axial load performed at time‐zero, which was used in all testing described, is a simplification of the complex loading conditions experienced in the knee during activities, and future studies should incorporate the dynamic loads through the knee during postoperative rehabilitation.

## CONCLUSION

Centralisation for various meniscal injuries associated with extrusion can reduce meniscal extrusion and improve joint biomechanics, along with clinical and radiological outcomes. Existing evidence is still scarce and exhibits a notable amount of methodological heterogeneity.

## AUTHOR CONTRIBUTIONS

**Khalis Boksh**: Conceptualization; methodology; formal analysis and investigation; writing, original draft. **Duncan Shepherd**: Writing—review and editing. **Daniel Espino**: Writing—review and editing. **Arijit Ghosh**: Methodology. **Randeep Aujla**: Methodology; formal analysis and investigation; writing—review and editing. **Tarek Boutefnouchet**: Conceptualization; methodology; writing—review and editing.

## CONFLICT OF INTEREST STATEMENT

The authors declare no conflict of interest.

## ETHICS STATEMENT

Ethical approval is not applicable for systematic reviews.

## Supporting information

Supporting information.

## Data Availability

Data are available from the corresponding author on request.

## References

[1] Amano, Y. , Ozeki, N. , Matsuda, J. , Nakamura, T. , Nakagawa, Y. , Sekiya, I. et al. (2023) Augmentation of a nonanatomical repair of a medial meniscus posterior root tear with centralization using three knotless anchors may be associated with less meniscal extrusion and better compressive load distribution in mid‐flexion compared with non‐anatomical root repair alone in a porcine knee model. Arthroscopy: The Journal of Arthroscopic & Related Surgery, 39(12), 2487–2498. Available from: 10.1016/j.arthro.2023.04.009 37142135

[2] Bedrin, M.D. , Kartalias, K. , Yow, B.G. & Dickens, J.F. (2021) Degenerative joint disease after meniscectomy. Sports Medicine and Arthroscopy Review, 29(3), e44–e50. Available from: 10.1097/JSA.0000000000000301 34398123

[3] Bhatia, S. , LaPrade, C.M. , Ellman, M.B. & LaPrade, R.F. (2014) Meniscal root tears: significance, diagnosis, and treatment. The American Journal of Sports Medicine, 42(12), 3016–3030. Available from: 10.1177/0363546514524162 24623276

[4] Boksh, K. , Ghosh, A. , Narayan, P. , Divall, P. & Aujla, R. (2023) Fibular versus tibiofibular‐based reconstruction of the posterolateral corner of the knee: a systematic review and meta‐analysis. The American Journal of Sports Medicine, 51(14), 3880–3892. Available from: 10.1177/03635465221138548 36598154

[5] Boksh, K. , Sheikh, N. , Chong, H.H. , Ghosh, A. & Aujla, R. (2024) The role of anterolateral ligament reconstruction or lateral extra‐articular tenodesis for revision anterior cruciate ligament reconstruction: a systematic review and meta‐analysis of comparative clinical studies. The American Journal of Sports Medicine, 52(1), 269–285. Available from: 10.1177/03635465231157377 36960926

[6] Chernchujit, B. & Agrawal, S. (2019) Arthroscopic all‐inside medial meniscus extrusion reduction. Arthroscopy Techniques, 8(5), e495–e501. Available from: 10.1016/j.eats.2019.01.008 31194115 PMC6551574

[7] Choi, S.H. , Bae, S. , Ji, S.K. & Chang, M.J. (2012) The MRI findings of meniscal root tear of the medial meniscus: emphasis on coronal, sagittal and axial images. Knee Surgery, Sports Traumatology, Arthroscopy, 20(10), 2098–2103. Available from: 10.1007/s00167-011-1794-4 22113225

[8] Chung, K.S. , Choi, C.H. , Bae, T.S. , Ha, J.K. , Jun, D.J. , Wang, J.H. et al. (2018) Comparison of tibiofemoral contact mechanics after various transtibial and all‐inside fixation techniques for medial meniscus posterior root radial tears in a porcine model. Arthroscopy: The Journal of Arthroscopic & Related Surgery, 34(4), 1060–1068. Available from: 10.1016/j.arthro.2017.09.041 29366743

[9] Chung, K.S. , Ha, J.K. , Ra, H.J. , Yu, W.J. & Kim, J.G. (2020) Root repair versus partial meniscectomy for medial meniscus posterior root tears: comparison of long‐term survivorship and clinical outcomes at minimum 10‐year follow‐up. The American Journal of Sports Medicine, 48(8), 1937–1944. Available from: 10.1177/0363546520920561 32437216

[10] Coleman, B.D. , Khan, K.M. , Maffulli, N. , Cook, J.L. & Wark, J.D. (2000) Studies of surgical outcome after patellar tendinopathy: clinical significance of methodological deficiencies and guidelines for future studies. Scandinavian Journal of Medicine & Science in Sports, 10(1), 2–11. Available from: 10.1034/j.1600-0838.2000.010001002.x 10693606

[11] Compagnoni, R. , Ferrua, P. , Minoli, C. , Fajury, R. , Ravaglia, R. , Menon, A. et al. (2023) The meniscal extrusion index is a reliable indirect sign of different meniscal lesion patterns: a classification based on percentage of meniscal extrusion. Knee Surgery, Sports Traumatology, Arthroscopy, 31(11), 5005–5011. Available from: 10.1007/s00167-023-07525-6 PMC10598112 37653144

[12] Costa, C.R. , Morrison, W.B. & Carrino, J.A. (2004) Medial meniscus extrusion on knee MRI: is extent associated with severity of degeneration or type of tear? American Journal of Roentgenology, 183(1), 17–23. Available from: 10.2214/ajr.183.1.1830017 15208101

[13] Crema, M.D. , Roemer, F.W. , Felson, D.T. , Englund, M. , Wang, K. , Jarraya, M. et al. (2012) Factors associated with meniscal extrusion in knees with or at risk for osteoarthritis: the Multicenter Osteoarthritis study. Radiology, 264(2), 494–503. Available from: 10.1148/radiol.12110986 22653191 PMC3401352

[14] Daney, B.T. , Aman, Z.S. , Krob, J.J. , Storaci, H.W. , Brady, A.W. , Nakama, G. et al. (2019) Utilization of transtibial centralization suture best minimizes extrusion and restores tibiofemoral contact mechanics for anatomic medial meniscal root repairs in a cadaveric model. The American Journal of Sports Medicine, 47(7), 1591–1600. Available from: 10.1177/0363546519844250 31091129

[15] Dean, R.S. , DePhillipo, N.N. , Monson, J.K. & LaPrade, R.F. (2020) Peripheral stabilization suture to address meniscal extrusion in a revision meniscal root repair: surgical technique and rehabilitation protocol. Arthroscopy Techniques, 9(8), e1211–e1218. Available from: 10.1016/j.eats.2020.04.022 32874903 PMC7451443

[16] Debieux, P. , Jimenez, A.E. , Novaretti, J.V. , Kaleka, C.C. , Kriscenski, D.E. , Astur, D.C. et al. (2021) Medial meniscal extrusion greater than 4 mm reduces medial tibiofemoral compartment contact area: a biomechanical analysis of tibiofemoral contact area and pressures with varying amounts of meniscal extrusion. Knee Surgery, Sports Traumatology, Arthroscopy, 29(9), 3124–3132. Available from: 10.1007/s00167-020-06363-0 33221933

[17] El‐Khoury, G.Y. , Usta, H.Y. & Berger, R.A. (1984) Meniscotibial (Coronary) ligamant tears. Skeletal Radiology, 11(3), 191–196. Available from: 10.1007/BF00349493 6547003

[18] Familiari, F. , Chahla, J. , Compagnoni, R. , DePhillipo, N.N.M , Moatshe, G. , LaPrade, R.F. et al (2024) Meniscal extrusion consensus statement: a collaborative survey within the Meniscus International Network (MenIN) Study Group. Knee Surgery, Sports Traumatology, Arthroscopy, 32(6), 1446–1454. Available from: 10.1002/ksa.12183 38606565

[19] Farivar, D. , Hevesi, M. , Fortier, L.M. , Azua, E. , LaPrade, R.F. & Chahla, J. (2023) Meniscal extrusion measurements after posterior medial meniscus root tears: a systematic review and meta‐analysis. The American Journal of Sports Medicine, 51(12), 3325–3334. Available from: 10.1177/03635465221131005 36541434

[20] Farivar, D. , Pascual, T.A. , Hevesi, M. & Chahla, J. (2024) Measuring technique for meniscal extrusion using ultrasound in the setting of posterior medial meniscal root tears. Arthroscopy Techniques, 13, 102916. Available from: 10.1016/j.eats.2024.102916 38690345 PMC11056738

[21] Foreman, S.C. , Liu, Y. , Nevitt, M.C. , Neumann, J. , Joseph, G.B. , Lane, N.E. et al. (2021) Meniscal root tears and extrusion are significantly associated with the development of accelerated knee osteoarthritis: data from the osteoarthritis initiative. Cartilage, 13(1), 239S–248S. Available from: 10.1177/1947603520934525 32567341 PMC8808926

[22] Gajjar, S.M. , Solanki, K.P. , Shanmugasundaram, S. & Kambhampati, S.B.S. (2021) Meniscal extrusion: a narrative review. Orthopaedic Journal of Sports Medicine, 9(11), 232596712110437. Available from: 10.1177/23259671211043797 PMC8573502 34778470

[23] Katagiri, H. , Nakagawa, Y. , Miyatake, K. , Ohara, T. , Shioda, M. , Sekiya, I. et al. (2023) Short‐term outcomes after high tibial osteotomy aimed at neutral alignment combined with arthroscopic centralization of medial meniscus in osteoarthritis patients. The Journal of Knee Surgery, 36(3), 261–268. Available from: 10.1055/s-0041-1731738 34261157

[24] Kijowski, R. , Woods, M.A. , McGuine, T.A. , Wilson, J.J. , Graf, B.K. & De Smet, A.A. (2011) Arthroscopic partial meniscectomy: MR imaging for prediction of outcome in middle‐aged and elderly patients. Radiology, 259(1), 203–212. Available from: 10.1148/radiol.11101392 21330563

[25] Kim, H. , Bin, S.I. , Kim, J.M. , Lee, B.S. & Sohn, D.W. (2021) Progression of allograft extrusion in both the coronal and sagittal planes at midterm follow‐up after medial meniscal allograft transplant. Orthopaedic Journal of Sports Medicine, 9(2), 232596712097235. Available from: 10.1177/2325967120972351 PMC7876759 33623794

[26] Kim, J.Y. , Bin, S.I. , Kim, J.M. , Lee, B.S. , Oh, S.M. , Cho, W.J. et al. (2020) Partial meniscectomy provides the favorable outcomes for symptomatic medial meniscus tear with an intact posterior root. Knee Surgery, Sports Traumatology, Arthroscopy, 28(11), 3497–3503. Available from: 10.1007/s00167-019-05634-9 31332494

[27] Kim, Y.M. , Joo, Y.B. , Noh, C.K. & Park, I.Y. (2016) The optimal suture site for the repair of posterior horn root tears: biomechanical evaluation of pullout strength in porcine menisci. Knee Surgery & Related Research, 28(2), 147–152. Available from: 10.5792/ksrr.2016.28.2.147 27274472 PMC4895087

[28] Koga, H. , Muneta, T. , Watanabe, T. , Mochizuki, T. , Horie, M. , Nakamura, T. et al. (2016) Two‐year outcomes after arthroscopic lateral meniscus centralization. Arthroscopy: The Journal of Arthroscopic & Related Surgery, 32(10), 2000–2008. Available from: 10.1016/j.arthro.2016.01.052 27132775

[29] Koga, H. , Muneta, T. , Yagishita, K. , Watanabe, T. , Mochizuki, T. , Horie, M. et al. (2012) Arthroscopic centralization of an extruded lateral meniscus. Arthroscopy Techniques, 1(2), e209–e212. Available from: 10.1016/j.eats.2012.08.001 23766997 PMC3678624

[30] Koga, H. , Nakamura, T. , Katagiri, H. , Nakagawa, Y. , Ozeki, N. , Ohara, T. et al. (2020) Two‐year outcomes after meniscoplasty by capsular advancement with the application of arthroscopic centralization technique for lateral compartment knee osteoarthritis. The American Journal of Sports Medicine, 48(12), 3154–3162. Available from: 10.1177/0363546520957367 33026837

[31] Koga, H. , Nakamura, T. , Nakagawa, Y. , Ozeki, N. , Ohara, T. , Shioda, M. et al. (2021) Arthroscopic centralization using knotless anchors for extruded medial meniscus. Arthroscopy Techniques, 10(3), e639–e645. Available from: 10.1016/j.eats.2020.10.051 33738196 PMC7953036

[32] Koga, H. , Watanabe, T. , Horie, M. , Katagiri, H. , Otabe, K. , Ohara, T. et al. (2017) Augmentation of the pullout repair of a medial meniscus posterior root tear by arthroscopic centralization. Arthroscopy Techniques, 6(4), e1335–e1339. Available from: 10.1016/j.eats.2017.05.014 29354437 PMC5622286

[33] Kohno, Y. , Koga, H. , Ozeki, N. , Matsuda, J. , Mizuno, M. , Katano, H. et al. (2022) Biomechanical analysis of a centralization procedure for extruded lateral meniscus after meniscectomy in porcine knee joints. Journal of Orthopaedic Research, 40(5), 1097–1103. Available from: 10.1002/jor.25146 34314533 PMC9292650

[34] Krych, A.J. , Bernard, C.D. , Leland, D.P. , Camp, C.L. , Johnson, A.C. , Finnoff, J.T. et al. (2020) Isolated meniscus extrusion associated with meniscotibial ligament abnormality. Knee Surgery, Sports Traumatology, Arthroscopy, 28(11), 3599–3605. Available from: 10.1007/s00167-019-05612-1 31332493

[35] Krych, A.J. , Boos, A.M. , Lamba, A. & Smith, P.A. (2024) Satisfactory clinical outcome, complications, and provisional results of meniscus centralization with medial meniscus root repair for the extruded medial meniscus at mean 2‐year follow‐up. Arthroscopy: The Journal of Arthroscopic & Related Surgery, 40(5), 1578–1587. Available from: 10.1016/j.arthro.2023.10.003 37832745

[36] Krych, A.J. , Nauert, R.F. , Song, B.M. , Cook, C.S. , Johnson, A.C. , Smith, P.A. et al. (2021) Association between transtibial meniscus root repair and rate of meniscal healing and extrusion on postoperative magnetic resonance imaging: a prospective multicenter study. Orthopaedic Journal of Sports Medicine, 9(8), 232596712110237. Available from: 10.1177/23259671211023774 PMC8371730 34423058

[37] Krych, A.J. , Song, B.M. , Nauert, R.F. , Cook, C.S. , Levy, B.A. , Camp, C.L. et al. (2022) Prospective consecutive clinical outcomes after transtibial root repair for posterior meniscal root tears: a multicenter study. Orthopaedic Journal of Sports Medicine, 10(2), 232596712210797. Available from: 10.1177/23259671221079794 PMC8882951 35237699

[38] Kubota, R. , Koga, H. , Ozeki, N. , Matsuda, J. , Kohno, Y. , Mizuno, M. et al. (2020) The effect of a centralization procedure for extruded lateral meniscus on load distribution in porcine knee joints at different flexion angles. BMC Musculoskeletal Disorders, 21, 205. Available from: 10.1186/s12891-020-03197-2 32245447 PMC7126455

[39] Kurosawa, H. , Fukubayashi, T. & Nakajima, H. (1980) Load‐bearing mode of the knee joint: physical behavior of the knee joint with or without menisci. Clinical Orthopaedics and Related Research, 149, 283–290. Available from: 10.1097/00003086-198006000-00039 7408313

[40] Leafblad, N.D. , Smith, P.A. , Stuart, M.J. & Krych, A.J. (2021) Arthroscopic centralization of the extruded medial meniscus. Arthroscopy Techniques, 10(1), e43–e48. Available from: 10.1016/j.eats.2020.09.005 33532206 PMC7823061

[41] Lerer, D.B. , Umans, H.R. , Hu, M.X. & Jones, M.H. (2004) The role of meniscal root pathology and radial meniscal tear in medial meniscal extrusion. Skeletal Radiology, 33(10), 569–574. Available from: 10.1007/s00256-004-0761-2 15316679

[42] Liberati, A. , Altman, D.G. , Tetzlaff, J. , Mulrow, C. , Gotzsche, P.C. , Ioannidis, J.P.A. et al. (2009) The PRISMA statement for reporting systematic reviews and meta‐analyses of studies that evaluate health care interventions: explanation and elaboration. BMJ, 339, b2700. Available from: 10.1136/bmj.b2700 19622552 PMC2714672

[43] Mass, H. & Katz, J.N. (2023) The influence of meniscal pathology in the incidence of knee osteoarthritis: a review. Skeletal Radiology, 52(11), 2045–2055. Available from: 10.1007/s00256-022-04233-z 36402862

[44] Mochizuki, Y. , Kawahara, K. , Samejima, Y. , Kaneko, T. , Ikegami, H. & Musha, Y. (2021) Short‐term results and surgical technique of arthroscopic centralization as an augmentation for medial meniscus extrusion caused by medial meniscus posterior root tear. European Journal of Orthopaedic Surgery & Traumatology, 31(6), 1235–1241. Available from: 10.1007/s00590-021-02874-9 33475853

[45] Morales‐Avalos, R. , Diabb‐Zavala, J.M. , Mohamed‐Noriega, N. , Vilchez‐Cavazos, F. , Perelli, S. , Padilla‐Medina, J.R. et al. (2023) Effect of injury to the lateral meniscotibial ligament and meniscofibular ligament on meniscal extrusion: biomechanical evaluation of the capsulodesis and centralization techniques in a porcine knee model. Orthopaedic Journal of Sports Medicine, 11, 23259671231212856. Available from: 10.1177/23259671231212856 38021298 PMC10668570

[46] Nishino, K. , Hashimoto, Y. , Iida, K. , Kinoshita, T. & Nakamura, H. (2023) Intrameniscal degeneration and meniscotibial ligament loosening are associated factors with meniscal extrusion of symptomatic discoid lateral meniscus. Knee Surgery, Sports Traumatology, Arthroscopy, 31(6), 2358–2365. Available from: 10.1007/s00167-022-07161-6 36112159

[47] Özdemir, M. & Turan, A. (2019) Correlation between medial meniscal extrusion determined by dynamic ultrasound and magnetic resonance imaging findings of medial‐type knee osteoarthritis in patients with knee pain. Journal of Ultrasound in Medicine, 38(10), 2709–2719. Available from: 10.1002/jum.14976 30828848

[48] Ozeki, N. , Koga, H. , Matsuda, J. , Kohno, Y. , Mizuno, M. , Katano, H. et al. (2020) Biomechanical analysis of the centralization procedure for extruded lateral menisci with posterior root deficiency in a porcine model. Journal of Orthopaedic Science, 25(1), 161–166. Available from: 10.1016/j.jos.2019.02.015 30902537

[49] Ozeki, N. , Muneta, T. , Kawabata, K. , Koga, H. , Nakagawa, Y. , Saito, R. et al. (2017) Centralization of extruded medial meniscus delays cartilage degeneration in rats. Journal of Orthopaedic Science, 22(3), 542–548. Available from: 10.1016/j.jos.2017.01.024 28351717

[50] Paletta, G.A. , Crane, D.M. , Konicek, J. , Piepenbrink, M. , Higgins, L.D. , Milner, J.D. et al. (2020) Surgical treatment of meniscal extrusion: a biomechanical study on the role of the medial meniscotibial ligaments with early clinical validation. Orthopaedic Journal of Sports Medicine, 8(7), 232596712093667. Available from: 10.1177/2325967120936672 PMC7391441 32775474

[51] Phillips, A.R. , Haneberg, E.C. , Boden, S.A. , Yanke, A.B. & Cole, B.J. (2024) Long term clinical and radiographic outcomes of meniscus allograft transplant. Current Reviews in Musculoskeletal Medicine. Available from: 10.1007/s12178-024-09904-z PMC11335704 38890265

[52] Randazzo, E. , Duerr, R. & Baria, M.R. (2022) Meniscus root tears: a clinical review. Current Sports Medicine Reports, 21(5), 155–158. Available from: 10.1249/JSR.0000000000000959 35522439

[53] Reisner, J.H. , Franco, J.M. , Hollman, J.H. , Johnson, A.C. , Sellon, J.L. & Finnoff, J.T. (2021) The difference in medial meniscal extrusion between non‐weight‐bearing and weight‐bearing positions in people with and without medial compartment knee osteoarthritis. PM&R, 13(5), 470–478. Available from: 10.1002/pmrj.12450 32652849

[54] Ro, K.H. , Kim, J.H. , Heo, J.W. & Lee, D.H. (2020) Clinical and radiological outcomes of meniscal repair versus partial meniscectomy for medial meniscus root tears: a systematic review and meta‐analysis. Orthopaedic Journal of Sports Medicine, 8(11), 232596712096207. Available from: 10.1177/2325967120962078 PMC7675875 33241058

[55] Saengpetch, N. , Noowan, S. , Boonrod, A. , Jaruwanneechai, K. , Sumanont, S. & Vijittrakarnrung, C. (2023) Comparison of medial tibiofemoral joint mechanics between all‑suture anchors and transtibial pullout technique for posterior medial meniscal root tears. Journal of Orthopaedic Surgery and Research, 18(1), 591. Available from: 10.1186/s13018-023-04071-2 37559157 PMC10413628

[56] Sterne, J.A. , Hernán, M.A. , Reeves, B.C. , Savović, J. , Berkman, N.D. , Viswanathan, M. et al. (2016) ROBINS‐I: a tool for assessing risk of bias in non‐randomised studies of interventions. BMJ, 355, i4919. Available from: 10.1136/bmj.i4919 27733354 PMC5062054

[57] Takase, R. , Ohsawa, T. , Hashimoto, S. , Kurihara, S. , Yanagisawa, S. , Hagiwara, K. et al. (2023) Insufficient restoration of meniscal extrusion by transtibial pullout repair for medial meniscus posterior root tears. Knee Surgery, Sports Traumatology, Arthroscopy, 31(11), 4895–4902. Available from: 10.1007/s00167-023-07528-3 37573532

[58] Ueki, H. , Kanto, R. , DiNenna, M. , Linde, M.A. , Fu, F.H. & Smolinski, P. (2023) Arthroscopic centralization reduces extrusion of the medial meniscus with posterior root defect in the ACL reconstructed knee. Knee Surgery, Sports Traumatology, Arthroscopy, 31(2), 543–550. Available from: 10.1007/s00167-022-07160-7 36114341

[59] Verdonk, P.C.M. , Verstraete, K.L. , Almqvist, K.F. , De Cuyper, K. , Veys, E.M. , Verbruggen, G. et al. (2006) Meniscal allograft transplantation: long‐term clinical results with radiological and magnetic resonance imaging correlations. Knee Surgery, Sports Traumatology, Arthroscopy, 14(8), 694–706. Available from: 10.1007/s00167-005-0033-2 16463170

[60] Walker, P.S. , Arno, S. , Bell, C. , Salvadore, G. , Borukhov, I. & Oh, C. (2015) Function of the medial meniscus in force transmission and stability. Journal of Biomechanics, 48(8), 1383–1388. Available from: 10.1016/j.jbiomech.2015.02.055 25888013

[61] Wang, M. , Bai, Y. , Sun, W. & Sun, J. (2023) Two years follow‐up of patients with knee varus deformity and medial meniscus extrusion after medial opening wedge high tibial osteotomy and arthroscopic meniscus centralization. International Orthopaedics, 48(2), 481–486. Available from: 10.1007/s00264-023-05959-8 37725151

[62] Winkler, P.W. , Wierer, G. , Csapo, R. , Hepperger, C. , Heinzle, B. , Imhoff, A.B. et al. (2020) Quantitative evaluation of dynamic lateral meniscal extrusion after radial repair. Orthopaedic Journal of Sports Medicine, 8(4), 232596712091456. Available from: 10.1177/2325967120914568 PMC7153201 32313812

