# Supplementary material for: Centralization reduces meniscal extrusion, improves joint mechanics and functional outcomes in patients undergoing meniscus surgery: A systematic review and meta‐analysis
Source: Knee Surg Sports Traumatol Arthrosc. 2024 Aug 9;33(3):888–906. doi: 10.1002/ksa.12410 (PMC11848986; doi:10.1002/ksa.12410)
Supplement: Supplementary file 1 — Supporting information. [file KSA-33-888-s001.docx]

|  |  |  |  |
| --- | --- | --- | --- |
|  | Pubmed (Medline) (14 July 2024) | EMBASE (14 July 2024) | Cochrane Central Register of Controlled Trials (14th July 2024) |
| 1 | centralization.mp | centralization.mp | centralization.mp |
| 2 | peripheral stabilization.mp | peripheral stabilization.mp | peripheral stabilization.mp |
| 3 | extrusion correction.mp | extrusion correction.mp | extrusion correction.mp |
| 4 | meniscotibial repair.mp | meniscotibial repair.mp | meniscotibial repair.mp |
| 5 | meniscus extrusion.mp | meniscus extrusion.mp | meniscus extrusion.mp |
| 6 | meniscus.mp | meniscus | meniscus |
| 7 | knee.mp | knee | knee |
|  | (1 OR 2 OR 3 OR 4) AND  (5 OR 6 OR 7) | (1 OR 2 OR 3 OR 4) AND  (5 OR 6 OR 7) | (1 OR 2 OR 3 OR 4) AND  (5 OR 6 OR 7) |
|  | Results: 533 | Results: 580 | Results: 166 |

**Online resource 1**. Search strategy for Pubmed (Medline), Embase and Cochrane Central Register of Controlled Trials


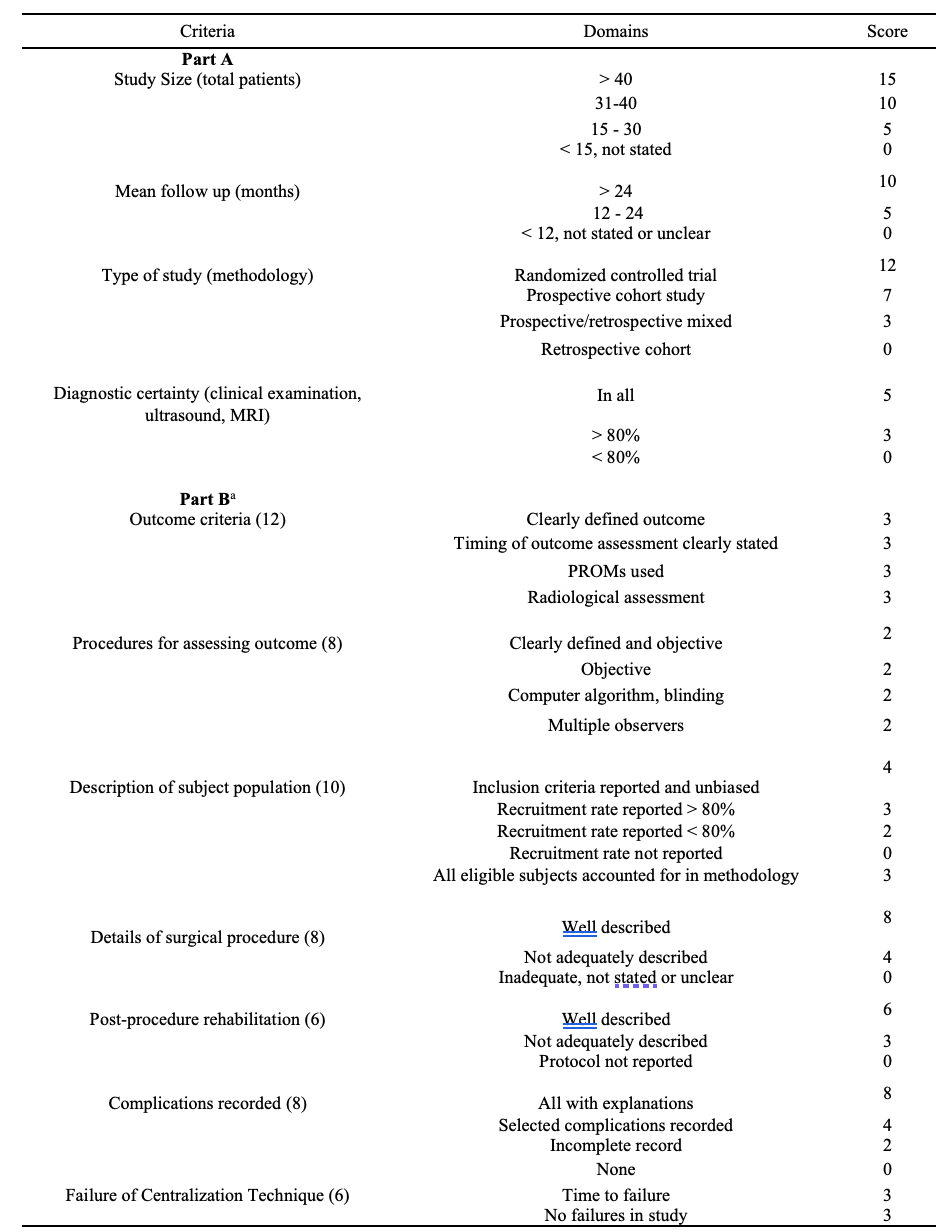


**Online resource 2.** Modified Coleman Criteria used for Assessment of the Quality of Clinical Studies

^a^ The values in parenthesis are total cumulative points

| Study | Meniscal Extrusion (mm) | | | | | |  |
| --- | --- | --- | --- | --- | --- | --- | --- |
|  | LMPRT/MMPRT vs Intact | LMPRT/MMPRT vs Centralization | Root repair vs intact | Root repair alone vs Root repair with centralization | Centralization vs Intact | Other comparisons |  |
| Daney 2019 [14] | **0°:** 1.83 ± 0.44 > 0.58 ± 0.44, p < 0.05 **90°**: 4.65 ± 0.46 > 1.84 ± 0.46, p < 0.05 | **ATPR + centralization 0°**: 1.83 ± 0.44 > -0.13 ± 0.44, p < 0.05 **90°**: 4.65 ± 0.46 > 1.77 ± 0.46, p < 0.05 **NA TPR + centralization 0°:** 1.83 ± 0.44 ~ 0.87 ± 0.44, p > 0.05 **90°**: 4.65 ± 0.46 > 3.26 ± 0.46, p < 0.05 | **Anatomic root repair 0°**: 0.70 ± 0.44 ~ 0.58 ± 0.44, p > 0.05 **90°:** 2.41 ± 0.46 ~ 1.84 ± 0.46, p > 0.05 **Non-anatomic root repair 0°:** 1.25 ± 0.44 ~ 0.58 ± 0.44, p > 0.05 **90°**: 3.29 ± 0.46 > 1.84 ± 0.46, p < 0.05 | **ATPR + centralization 0°:** 0.70 ± 0.44 (ATPR) ~ -0.13 ± 0.44, p > 0.05 **90°:** 2.41 ± 0.46 (ATPR) ~ 1.77 ± 0.46, p > 0.05 **NATPR + centralization** **0°:** 1.25 ± 0.44 (NATPR) ~ 0.87 ± 0.44, p > 0.05 **90°**: 3.29 ± 0.46 (NATPR) ~ 3.26 ± 0.46,  p > 0.05 | **ATPR + centralization  0°:** -0.13 ± 0.44 ~ 0.58 ± 0.44, p > 0.05 **90°:** 1.77 ± 0.46 ~ 1.84 ± 0.46, p > 0.05 **NA TPR + centralization 0°**: 0.87 ± 0.44 ~ 0.58 ± 0.44, p > 0.05 **90°**: 3.26 ± 0.46 > 1.84 ± 0.46, p < 0.05 | N/A |  |
| Ozeki 2020 [48] | 21.9 ± 2.0 > 18.1 ± 1.9, p = 0.031 | 21.9 ± 2.0 > 15.3 ± 1.3, p = 0.031 | N./A | N/A | 15.3 ± 1.3 ~ 18.1 ± 1.9, p = 0.31 | N/A |  |
| Kubota 2020 [38] | **30°**: 22.2 ± 1.3 > 16.7 ± 1.3, p < 0.05 **45°:** 24.0 ± 1.0 > 20.0 ± 1.1, p < 0.05 **60°:** 25.8 ± 1.8 > 19.3 ± 0.7, p < 0.05 **90°:** 27.6 ± 1.8 > 20.2 ± 1.1, p < 0.05 | **30°**: 22.2 ± 1.3 > 15.5 ± 1.5, p < 0.05 **45°:** 24.0 ± 1.0 > 15.8 ± 1.3, p < 0.05 **60°:** 25.8 ± 1.8 > 17.9 ± 1.3, p < 0.05 **90°**: 27.6 ± 1.8 > 20.2 ± 1.8, p < 0.05 | N/A | N/A | **30°**: 15.5 ± 1.5 ~ 16.7 ± 1.3, p > 0.05 **45°**: 15.8 ± 1.3 ~ 20.0 ± 1.1, p > 0.05 **60°:** 17.9 ± 1.3 ~ 19.3 ± 0.7, p > 0.05 **90°**: 20.2 ± 1.8 ~ 20.2 ± 1.1, p > 0.05 | N/A |  |
| Paletta Jr 2020 [50] | N/A | N/A | N/A | N/A | **0°:** 2.1 ± 0.4 vs 1.5 ± 0.6, p > 0.05 | **Intact vs MMTL injury** **0°**: 1.5 ± 0.6 < 3.4 ± 0.7,  p < 0.05 **Centralization vs MMTL injury** **0°:** 2.1 ± 0.4 < 3.4 ± 0.7, p < 0.05 |  |
| Debieux 2020 [16] | N/A | N/A | N/A | N/A | Centralization ~ Intact, p > 0.05 | N/A |  |
| Kohno 2022 [33] | Not measured | Not measured |  | N/A | Not measured | N/A |  |
| Amano 2023 [1] | **30°**: 2.4 ± 0.53 > 0.25 ± 0.19, p < 0.05 **45°**: 2.7 ± 0.55 > 0.77 ± 0.21, p < 0.05 **60°**: 3.4 ± 0.58 > 0.76 ± 0.17, p < 0.05 **90°**: 3.8 ± 1.2 > 0.67 ± 0.16, p < 0.05 | **2-anchor centralization 30°**: 2.4 ± 0.53 > 1.2 ± 0.52 p < 0.05 **45°**: 2.7 ± 0.55 > 1.3 ± 0.55, p < 0.05 **60°**: 3.4 ± 0.58 > 1.6 ± 0.63, p < 0.05 **90°**: 3.8 ± 1.2 > 2.1 ± 0.65, p < 0.05 **3-anchor centralization**  **30°**: 2.4 ± 0.53 > -0.06 ± 0.73 p < 0.05 **45°**: 2.7 ± 0.55 > 0.21 ± 0.75, p < 0.05 **60°**: 3.4 ± 0.58 > 0.78 ± 0.87, p < 0.05 **90°**: 3.8 ± 1.2 > 1.1 ± 0.93, p < 0.05 | **30°**: 1.5 ± 0.47 ~ 0.25 ± 0.19, p > 0.05 **45°**: 1.7 ± 0.46 ~ 0.77 ± 0.21, p > 0.05 **60°**: 2.3 ± 0.4 ~ 0.76 ± 0.17, p > 0.05 **90°**: 2.2 ± 0.53 ~ 0.67 ± 0.16, p > 0.05 | **2-anchor centralization 30°:** 1.5 ± 0.47 ~ 1.2 ± 0.52 p > 0.05 **45°:** 1.7 ± 0.46 ~ 1.3 ± 0.55, p > 0.05 **60°:** 2.3 ± 0.4 ~ 1.6 ± 0.63, p > 0.05 **90°:** 2.2 ± 0.53 ~ 2.1 ± 0.65, p > 0.05 **3-anchor centralization  30°:** 1.5 ± 0.47 > -0.063 ± 0.73 p < 0.05 **45°:** 1.7 ± 0.46 > 0.21 ± 0.75, p < 0.05 **60°:** 2.3 ± 0.4 > 0.78 ± 0.87, p < 0.05 **90°:** 2.2 ± 0.53 ~ 1.1 ± 0.93, p > 0.05 | **2-anchor centralization** 30°: 1.2 ± 0.52 ~ 0.25 ± 0.19, p > 0.05 45°: 1.3 ± 0.55 ~ 0.77 ± 0.21, p > 0.05 60°: 1.6 ± 0.63 ~ 0.76 ± 0.17, p > 0.05 90°: 2.1 ± 0.65 ~ 0.67 ± 0.16, p > 0.05 **3-anchor centralization**  30°:-0.06 ± 0.73 ~ 0.25 ± 0.19, p > 0.05 45°: 0.21 ± 0.75 ~ 0.77 ± 0.21, p > 0.05 60°: 0.78 ± 0.87 ~ 0.76 ± 0.17, p > 0.05 90°: 1.1 ± 0.93 ~ 0.67 ± 0.16, p > 0.05 | N/A |  |
|  |  |  |  |  |  |  |  |
| Morales-Avalos 2023 [45] | N/A | N/A | N/A | N/A | **30°**: 1.42 ± 0.1 ~ 1.66 ± 0.65, p > 0.05 **60°:** 1.47 ± 0.05 ~ 1.69 ± 0.07, p > 0.05 | **Centralization vs LMTL injury  30°**: 1.42 ± 0.1 < 2.11 ± 0.16, p < 0.05 **60°**: 1.47 ± 0.05 < 2.42 ± 0.17, p < 0.05 |  |
| Ueki 2023 [58] | **0°**: 2.4 ± 1.4 > 0.6 ± 0.6, p < 0.05 **30°**: 2.4 ± 0.9 > 0.6 ± 0.5, p < 0.05 | **0°**: 2.4 ± 1.4 > 0.75 ± 0.7, p < 0.05 **30°**: 2.4 ± 0.9 > 0.85 ± 0.63, p < 0.05 | N/A | N/A | **0°**: 0.75 ± 0.7 ~ 0.6 ± 0.6, p > 0.05 **30°**: 0.85 ± 0.63 ~ 0.6 ± 0.5, p > 0.05 | N/A |  |

**Online resource 3.** Qualitative synthesis of the differences in meniscal extrusion in various testing state conditions. ATPR, anatomical transtibial pull-through repair; MMPRT, medial meniscus posterior root tear; LMPRT; lateral meniscus posterior root tear; LMTL, lateral meniscotibial ligament; MMTL, medial meniscotibial ligament; NA, not applicable; NATPR, non-anatomical transtibial pull-through repair

| Study | Average contact pressure (MPa) | | | | | |  |
| --- | --- | --- | --- | --- | --- | --- | --- |
|  | LMPRT/MMPRT vs Intact | LMPRT/MMPRT vs Centralization | Root repair vs intact | Root repair alone vs Root repair with centralization | Centralization vs Intact | Other comparisons |  |
| Daney 2019 [14] | **0, 30, 60 and 90°** Root tear < Intact, p < 0.05 | **0, 30, 60 and 90°** Root tear < ATPR + centralization **0, 30 and 90°** Root tear ~ NATPR + centralization  **60°:** Root tear < NATPR + centralization | **0, 30 and 90°** ATPR/NATPR ~ Intact,  p > 0.05 **60°**  ATPR ~ Intact, p > 0.05 NATPR < Intact,  p < 0.05 | **0, 30, 60 and 90°** ATPR ~ ATPR + centralization, p > 0.05 NATPR ~ NATPR + centralization, p > 0.05 | **0 and 30°** ATPR+C ~ Intact, p > 0.05 NATPR+C~ Intact, p>0.05 **60° and 90°** ATPR+C ~ Intact, p > 0.05 NATPR +C < Intact, p<0.05 |  |  |
| Ozeki 2020 [48] | **45°.** TC: 0.153 ± 0.02 > 0.09 ± 0.02, p = 0.004 | **45°.** TC: 0.153 ± 0.02 ~ 0.102 ± 0.43, p > 0.05 |  |  | **45°.** TC: 0.102 ± 0.43 ~ 0.09 ± 0.02, p > 0.05 |  |  |
| Kubota 2020 [38] | **30°.** TC: 0.133 ± 0.01 ~ 0.09 ± 0.02, p > 0.05  **45°.** TC: 0.17 ± 0.014 > 0.095 ± 0.01, p < 0.05 **60°.** TC: 0.168 ± 0.012 > 0.095 ± 0.008, p < 0.05 **90°.** TC: 0.161 ± 0.009 > 0.068 ± 0.007, P < 0.05 | **30°.** TC: 0.133 ± 0.01 ~ 0.125 ± 0.01, p > 0.05 **45°.** TC: 0.17 ± 0.014 ~ 0.132 ± 0.012, p > 0.05 **60°.** TC: 0.168 ± 0.012 ~ 0.118 ± 0.01, p > 0.05 **90°**.TC: 0.161 ± 0.009 > 0.095 ± 0.02, p < 0.05 |  |  | **30°.** TC: 0.125 ± 0.01 ~ 0.09 ± 0.02, p > 0.05  **45°.** TC: 0.132 ± 0.012 ~ 0.095 ± 0.01, p > 0.05 **60°.** TC: 0.118 ± 0.01 ~ 0.095 ± 0.008, p > 0.05 **90°.** TC: 0.095 ± 0.02 ~ 0.068 ± 0.007, p > 0.05 | N/A |  |
| Paletta Jr 2020 [50] | N/A | N/A | N/A | N/A | N/A | N/A |  |
| Debieux 2020 [16] | N/A | N/A | N/A | N/A | **0, 30, 60 and 90°** C ~ Intact, p > 0.05 | **0, 30, 60 and 90°** C ~ Maximum Extrusion, p > 0.05 Intact ~ Maximum Extrusion, p > 0.05 |  |
| Kohno 2022 [33] | **45°.** TC: 0.23 ± 0.1 > 0.085 ± 0.02, p < 0.05 | **45° 1-anchor centralization** TC: 0.23 ± 0.1 ~ 0.21 ± 0.02, p > 0.05 **2-anchor centralization** TC: 0.23 ± 0.1 ~ 0.16 ± 0.02, p > 0.05 **Centralization with advancement**  TC: 0.23 ± 0.1 > 0.11 ± 0.03, p < 0.05 |  |  | **45° 1-anchor centralization** TC: 0.21 ± 0.02 > 0.085 ± 0.02, p < 0.05 **2-anchor centralization** TC: 0.16 ± 0.02 ~ 0.085 ± 0.02, p > 0.05 **Centralization with advancement**  TC: 0.11 ± 0.03 ~ 0.085 ± 0.02, p > 0.05 | **Centralization vs. PM at 45° 1-anchor centralization** TC: 0.21 ± 0.02 ~ 0.14 ± 0.02, p > 0.05 **2-anchor centralization** TC: 0.16 ± 0.02 ~ 0.14 ± 0.02, p > 0.05 **Centralization with advancement** TC: 0.11 ± 0.03 ~ 0.14 ± 0.02, p > 0.05 |  |
| Amano 2023 [1] | **30°.** TC: 1.2 ± 0.08 > 0.53 ± 0.12, p < 0.05 **45°.** TC: 1.3 ± 0.05 > 0.48 ± 0.13, p < 0.05 **60°.** TC: 1.3 ± 0.05 > 0.35 ± 0.09, p < 0.05 **90°.** TC: 1.3 ± 0.05 > 0.31 ± 0.09, p < 0.05 | **2-anchor centralization 30°.** TC: 1.2 ± 0.08 > 0.60 ± 0.13, p < 0.05 **45°.** TC: 1.3 ± 0.05 > 0.72 ± 0.13, p < 0.05 **60°.** TC: 1.3 ± 0.05 > 0.63 ± 0.11, p < 0.05 **90°.** TC: 1.3 ± 0.05 > 0.59 ± 0.09, p < 0.05 **3-anchor centralization 30°.** TC: 1.2 ± 0.08 > 0.42 ± 0.09, p < 0.05 **45°.** TC: 1.3 ± 0.05 > 0.49 ± 0.12, p < 0.05 **60°.** TC: 1.3 ± 0.05 > 0.54 ± 0.11, p < 0.05 **90°.** TC: 1.3 ± 0.05 > 0.49 ± 0.14, p < 0.05 | **30°**. TC: 0.83 ± 0.08 > 0.53 ± 0.12, p < 0.05 **45°.** TC: 0.84 ± 0.12 > 0.48 ± 0.13, p < 0.05 **60°.** TC: 0.74 ± 0.11 > 0.35 ± 0.09, p < 0.05 **90°.** TC: 0.75 ± 0.10 > 0.31 ± 0.09, p < 0.05 | **2-anchor centralization 30°.** TC: 0.83 ± 0.08 > 0.60 ± 0.13, p < 0.05 **45°.** TC: 0.84 ± 0.12 ~ 0.72 ± 0.13, p > 0.05 **60°.** TC: 0.74 ± 0.11 ~ 0.63 ± 0.11, p > 0.05 **90°.** TC: 0.75 ± 0.10 ~ 0.59 ± 0.09, p > 0.05 **3-anchor centralization 30°.** TC: 0.83 ± 0.08 > 0.42 ± 0.09, p < 0.05 **45°.** TC: 0.84 ± 0.12 > 0.49 ± 0.12, p < 0.05 **60°.** TC: 0.74 ± 0.11 > 0.54 ± 0.11, p < 0.05 **90°.** TC: 0.75 ± 0.10 > 0.49 ± 0.14, p < 0.05 | **2-anchor centralization 30°.** TC: 0.60 ± 0.13 ~ 0.53 ± 0.12, p > 0.05 **45°.** TC: 0.72 ± 0.13 ~ 0.48 ± 0.13, p > 0.05 **60°.** TC: 0.63 ± 0.11 > 0.35 ± 0.09, p < 0.05 **90°.** TC: 0.59 ± 0.09 > 0.31 ± 0.09, p < 0.05 |  |  |
|  |  |  |  |  |  |  |  |
| Ueki 2023 [58] | N/A | N/A | N/A | N/A | N/A | N/A |  |
| Morales-Avalos 2023 [45] | N/A | N/A | N/A | N/A | N/A | N/A |  |

**Online resource 4.**  Qualitative synthesis of the differences in average contact pressure in various testing state conditions. ATPR, anatomical transtibial pull-through repair; C, centralization; MMPRT, medial meniscus posterior root tear; LMPRT; lateral meniscus posterior root tear; N/A, not applicable; NATPR, non-anatomical transtibial pull-through repair; TC, tibial cartilage.

| Study | Average contact area (mm^2^) | | | | | |  |
| --- | --- | --- | --- | --- | --- | --- | --- |
|  | LMPRT/MMPRT vs Intact | LMPRT/MMPRT vs Centralization | Root repair vs intact | Root repair alone vs Root repair with centralization | Centralization vs Intact | Other comparisons |  |
| Daney 2019 [14] | **0, 30, 60 and 90°** Root tear < Intact, p < 0.05 | **0° and 30°** Root tear ~ Centralization, p > 0.05 **60° and 90°** Root tear < ATPR + centralization, p < 0.05 Root tear < NATPR + centralization, p < 0.05 | **0° and 30°** ATPR/NATPR ~ Intact, p > 0.05 **60° and 90°** ATPR ~ Intact, p > 0.05 NATPR < Intact. P < 0.05 | **0, 30, 60 and 90°** ATPR ~ ATPR + C,  p > 0.05 NATPR ~ NATPR + C,  p > 0.05 | **0°:** ATPR + C ~ Intact, p > 0.05 NATPR + C ~ Intact, p > 0.05 **30° and 90°** ATPR + C ~ Intact, p > 0.05 NATPR + C < Intact, p < 0.05  **60°:** ATPR+ C < Intact, p < 0.05 NATPR + C < Intact, p < 0.05 | N/A |  |
| Ozeki 2020 [48] | **45°.** MB: 45.8 ± 13.7 < 85.7 ± 6.8, p = 0.013 | **45°.** MB: 45.8 ± 13.7 < 98.3 ± 4.75, p = 0.005 |  |  | **45°.** MB: 98.3 ± 4.75 ~ 85.7 ± 6.8, p > 0.05 | N/A |  |
| Kubota 2020 [38] | **30°**. MB: 13.8 ± 6.63 < 70.9 ± 9.55, p < 0.05 **45°**. MB: 3.1 ± 1.3 < 93.1 ± 6.45, p < 0.05 **60°**. MB: 12.0 ± 8.45 < 82.8 ± 6.73, p < 0.05 **90°.** MB: 8.6 ± 3.9 < 72.9 ± 7.2 p < 0.05 | **30°.** MB: 13.8 ± 6.63 < 66.9 ± 9.1, p < 0.05 **45°.** MB: 3.1 ± 1.3 < 86.0 ± 8.0, p < 0.05 **60°.** MB: 12.0 ± 8.45 < 90.6 ± 6.05, p < 0.05 **90°**. MB: 8.6 ± 3.9 < 78.0 ± 9.4, p < 0.05 | N/A | N/A | **30°**. MB: 66.9 ± 9.1 ~ 70.9 ± 9.55, p > 0.05 **45°.** MB: 86.0 ± 8.0 ~ 93.1 ± 6.45, p > 0.05 **60°.** MB: 90.6 ± 6.05 ~ 82.8 ± 6.73, p > 0.05 **90°**. MB: 78.0 ± 9.4 ~ 72.9 ± 7.2 p > 0.05 | N/A |  |
| Paletta Jr 2020 [50] | N/A | N/A | N/A | N/A | N/A | N/A |  |
| Debieux 2020 [16] | N/A | N/A | N/A | N/A | **0°**: 427 **±** 144 ~ 456 ± 102, p > 0.05 **30° :** 425 ± 157 ~ 544 ± 120, p > 0.05 **60° :** 380 ± 140 ~ 429 ± 125, p > 0.05 **90° :** 283 ± 148 < 382 ± 124, p < 0.05 | **Centralization vs. Maximum extrusion** **0°** : 427 ± 144 ~ 360 ± 146, p > 0.05 **30°** : 425 ± 157 > 385 ± 140, p < 0.05 **60°** : 380 ± 140 > 304 ± 127, p < 0.05 **90°** : 283 ± 148 ~ 238 ± 148, p > 0.05 **Intact vs. Maximum extrusion** 0°: 456 ± 102 > 360 ± 146, p < 0.05 30° : 544 ± 120 > 385 ± 140, p < 0.05 60° : 429 ± 125 > 304 ± 127, p < 0.05 90° : 382 ± 124 > 238 ± 148, p < 0.05 |  |
| Kohno 2022 [33] | **45°** MB: 10.7 ± 9.68 < 104.8 ± 6.13, p < 0.05 | **45° 1-anchor centralization** MB: 10.7 ± 9.68 ~ 59.2 ± 9.9, p > 0.05 **2-anchor centralization**  MB: 10.7 ± 9.68 ~ 76.3 ± 6.08, p > 0.05 **Centralization with advancement**  MB: 10.7 ± 9.68 < 87.7 ± 4.13, p < 0.05 | N/A | N/A | **45° 1-anchor centralization** MB: 59.2 ± 9.9 < 104.8 ± 6.13 p < 0.05 **2-anchor centralization**  MB: 76.3 ± 6.08 ~ 104.8 ± 6.13, p > 0.05 **Centralization with advancement**  MB: 87.7 ± 4.13 ~ 104.8 ± 6.13, p >0.05 | **Centralization vs. PM at 45° 1-anchor centralization** MB: 59.2 ± 9.9 ~ 74.7 ± 4.85, p > 0.05 **2-anchor centralization**  MB: 76.3 ± 6.08 ~ 74.7 ± 4.85, p > 0.05 **Centralization with advancement**  MB: 87.7 ± 4.13 ~ 74.7 ± 4.85, p > 0.05 |  |
| Amano 2023 [1] | **30°.** MB: 1.3 ± 1.3 < 71.7 ± 10.6, p < 0.05 **45°.** MB: 0.72 ± 0.65 < 98.1 ± 10.8, p < 0.05 **60°.** MB: 0 ± 0 < 110.1 ± 9.98, p < 0.05 **90°.** MB: 0.08 ± 0.08 < 94.6 ± 11, p < 0.05 | **2 anchor centralization 30°.** MB: 1.3 ± 1.3 < 68.6 ± 10.1, p < 0.05 **45°.** MB: 0.72 ± 0.65 < 76.6 ± 12.3, p < 0.05 **60°.** MB: 0 ± 0 < 61.4 ± 13.2, p < 0.05 **90°.** MB: 0.08 ± 0.08 < 45.6 ± 10.3, p < 0.05 **3 anchor centralization 30°.** MB: 1.3 ± 1.3 < 86.4 ± 8.1, p < 0.05 **45°.** MB: 0.72 ± 0.65 < 98.1 ± 7.95, p < 0.05 **60°.** MB: 0 ± 0 < 76.1 ± 9.73, p < 0.05 **90°.** MB: 0.08 ± 0.08 < 62.4 ± 12.6, p < 0.05 | **30°.** MB: 33.3 ± 6 < 71.7 ± 10.6, p < 0.05 **45°.**MB: 35.1 ± 7 < 98.1 ± 10.8, p < 0.05 **60°.** MB: 38.5 ± 6.73 < 110.1 ± 9.98, p < 0.05 **90°.** MB: 27.1 ± 7.95 < 94.6 ± 11, p < 0.05 | **2 anchor centralization 30°.** MB: 33.3 ± 6 < 68.6 ± 10.1, p < 0.05 **45°.** MB: 35.1 ± 7 < 76.6 ± 12.3, p < 0.05 **60°.** MB: 38.5 ± 6.73 < 61.4 ± 13.2, p < 0.05 **90°.** MB: 27.1 ± 7.95 ~ 45.6 ± 10.3, p > 0.05 **3 anchor centralization 30°.** MB: 33.3 ± 6 < 86.4 ± 8.1, p < 0.05 **45°.** MB: 35.1 ± 7 < 98.1 ± 7.95, p < 0.05 **60°.** MB: 38.5 ± 6.73 < 76.1 ± 9.73, p < 0.05 **90°.** MB: 27.1 ± 7.95 < 62.4 ± 12.6, p < 0.05 | **2 anchor centralization 30°.** MB: 68.6 ± 10.1 ~ 71.7 ± 10.6, p > 0.05 **45°.** MB: 76.6 ± 12.3 ~ 98.1 ± 10.8, p > 0.05 **60°.** MB: 61.4 ± 13.2 < 110.1 ± 9.98, p < 0.05 **90°.** MB: 45.6 ± 10.3 < 94.6 ± 11, p < 0.05 **3 anchor centralization 30°.** MB: 86.4 ± 8.1 ~ 71.7 ± 10.6, p > 0.05 **45°.** MB: 98.1 ± 7.95 ~ 98.1 ± 10.8, p > 0.05 **60°.** MB: 76.1 ± 9.73 ~ 110.1 ± 9.98, p > 0.05 **90°.** MB: 62.4 ± 12.6 < 94.6 ± 11, p < 0.05 | **N/A** |  |
|  |  |  |  |  |  |  |  |
| Ueki 2023 [58] | N/A | N/A | N/A | N/A | N/A | N/A |  |
| Morales-Avalos 2023 [45] | N/A | N/A | N/A | N/A | N/A | N/A |  |

**Online resource 5.** Qualitative synthesis of the differences in average contact area (mm^2^) in various testing state conditions. ATPR, anatomical transtibial pull-through repair; C, centralization; MB, midbody of meniscus; MMPRT, medial meniscus posterior root tear; LMPRT; lateral meniscus posterior root tear; N/A, not applicable; NATPR, non-anatomical transtibial pull-through repair

| Study | Lysholm | KOOS | Definition of Meniscal Extrusion | Meniscal Extrusion (mm) |
| --- | --- | --- | --- | --- |
| Koga 2016 [28] | **Post-op > Pre-op**  96.5 ± 3.3 vs 69.2 ± 14.5 , p < 0.05 | **Post-op > Pre-op**  Pain: 88.9 ± 9.8 vs 72.2 ± 6.8, p < 0.05 Symptoms: 91.1 ± 6.6 vs 74.4 ± 9.7, p < 0.05 Sports: 78.8 ± 23.8 vs 41.7 ± 18.9, p < 0.05 QoL: 78.1 ± 17.5 vs 45.8 ± 25.8, p < 0.05  Post-op ~ Pre-op  ADLs: 94.4 ± 7.4 vs 88.5 ± 5.7, p > 0.05 | Distance (mm) from peripheral aspect of meniscus to border of tibia, excluding osteophytes | **At 1 year Pre-op > post-op for LM defect** 5.0 ± 1.5 vs 1.0 ± 0.75, p < 0.05 **Pre-op > post-op for discoid LM**  1.6 ± 1.25 > 0.3 ± 0.75, p < 0.05 |
| Paletta Jr 2020 [50] | N/A | N/A | Distance (mm) from tibial plateau to outer edge of medial meniscus | **Pre-op > Post-op** 2.4 ± 0.5 > 1.2 ± 0.6, p < 0.05 |
| Koga 2020 [30] | **Post-op > Pre-op** 92.8 ± 6.4 vs 64.9 ± 13.9,  p < 0.05 | **Post-op > Pre-op**  Pain: 88.5 ± 10.7 vs 64.9 ± 19.1, p < 0.05 Symptoms: 81.6 ± 11.5 vs 61.4 ± 17.4, p < 0.05 ADLs: 95.5 ± 4.5 vs 79.1 ± 17.5, p < 0.05 Sports: 72.9 ± 20.3 vs 38.7 ± 24.8, p < 0.05 QoL: 69.7 ± 19.3 vs 36.5 ± 20.7, p < 0.05 | Distance (mm) from peripheral aspect of meniscus to border of tibia, excluding osteophytes | **At 1 year Pre-op > Post-op** 4.9 ± 1.25 vs 1.1 ± 0.75, p < 0.05 |
| Mochizuki 2021 [44] | **Post-op > Pre-op**  78.5 ± 7.9 vs 46.0 ± 8.8,  p < 0.05 | **Post-op > Pre-op**  Pain: 81.1 ± 6.1 vs 47.4 ± 10.9, p < 0.05 Symptoms: 81.0 ± 9.7 vs 53.2 ± 10.8, p < 0.05 ADLs: 86.3 ± 6.6 vs 64.6 ± 10.5, p < 0.05 Sports: 66.4 ± 5.8 vs 18.9 ± 6.1, p < 0.05 QoL: 68.2 ± 13.6 vs 34.9 ± 11.4, p < 0.05 | Distance (mm) from peripheral aspect of meniscus to border of medial tibial plateau | **Pre-op > Post-op** 4.8 ± 0.7 vs 2.7 ± 0.3, p < 0.05 |
| Katagiri 2023 [23] | **OWHTO + centralization ~ OWHTO** 94.1 ± 1.3 vs 93.3 ± 2.4, p > 0.05 | **OWHTO + centralization ~ OHWTO** Pain: 82.9 ± 3.6 vs 82.7 ± 5.4, p > 0.05 Symptoms: 81.8 ± 3.4 vs 82.2 ± 5.1, p > 0.05 ADLs: 90.3 ± 2.7 vs 93.2 ± 2.5, p > 0.05 Sports: 67.1 ± 5.6 vs 67.9 ± 8.9, p > 0.05 QoL: 67.9 ± 5.6 vs 73.7 ± 6.6, p > 0.05 | N/A | N/A |
| Wang 2023 [61] | N/A | N/A | Furthest edge of MM to most lateral edge of MTP | N/A |
| Krych 2023 [35] | N/A | **KOOS Jr  post-op > Pre-op** 81.3 ± 12.8 vs 58.2 ± 9.3, p < 0.05 | N/A | Not enough data (3 patients) |

**Online resource 6.** Qualitative synthesis of Lysholm, KOOS and Meniscal Extrusion following meniscus centralization. ADL, activities of daily living; KOOS, Knee Injury and Osteoarthritis Outcome Score; LM, lateral meniscus; MM, medial meniscus; MTP, medial tibial plateau; NA, not applicable; OWHTO, open wedge high tibial osteotomy; QOL, quality of life

| IKDC | Knee Examination  (Difference between injured and non-injured knee) | | Joint space width (mm) on x-ray | Complications |  |  |
| --- | --- | --- | --- | --- | --- | --- |
| Study |  |  | Extension |  | Flexion |  |
| Koga 2016 [28] | N/A | **Post-op > Pre-op** 0.1 ± 0.3 vs 1.5 ± 3.1, p = 0.05 | **Post-op > Pre-op** 0.5 ± 1.5 vs 4.0 ± 10.1, p = 0.01 | **Post-op > pre-op for LM defect** 5.6 ± 0.75 vs 4.8 ± 1.25, p < 0.05 **Post-op ~ pre-op for discoid LM**  5.5 ± 1.25 vs 5.4 ± 1.25, p > 0.05 | 0 |  |
| Paletta Jr [50] | N/A | N/A | N/A | N/A | NR |  |
| Koga 2020 [30] | **Post-op > Pre-op** 75.8 ± 12.6 vs 51.8 ± 15.0, p < 0.05 | **Post-op > Pre-op** 2.2 ± 3.8 vs.1.9 ± 3.1, p = 0.178 | **Post-op > Pre-op** 0.1 ± 0.4 vs 4.3 ± 8.2, p = 0.216 | **Post-op > pre-op** 3.3 ± 1.25 vs 2.1 ± 1, p < 0.05 | 1 ->persistent pain-> Distal femoral osteotomy |  |
| Mochizuki 2021[44] | N/A | N/A | N/A | N/A | 2->Total knee replacement |  |
| Katagiri 2023 [23] | **OWHTO + centralization ~ OWHTO** 71.8 ± 3.95 vs 65.5 ± 5.0, p > 0.05 | N/A | N/A | **OWHTO + Centralization:** **Post-op>pre-op** 2.7 ± 0.13 vs 1.9 ± 0.25, p < 0.05 **OWHTO: Post-op ~ pre-op** 2.2 ± 0.33 vs 2.2 ± 0.43, p > 0.05 | 0 |  |
| Wang 2023 [61] | N/A | N/A | N/A | N/A | 2->Surgical site infection 2-> stiffness |  |
| Krych 2023 [35] | **Post-op > Pre-op** 70.4 ±16.8 vs 46.3 ± 10.8, p < 0.05 | N/A | N/A | N/A | 1-> arthrofibrosis |  |

**Online resource 7.** Qualitative synthesis of IKDC score, knee range of motion, joint space width on x-rays and post-operative complications. IKDC, international knee documentation committee; N/A, not applicable; NR, not reported; OWHTO, open wedge high tibial osteotomy
